# Ti_3_C_2_T_*x*_ MXenes-based flexible materials for electrochemical energy storage and solar energy conversion

**DOI:** 10.1515/nanoph-2022-0228

**Published:** 2022-06-09

**Authors:** Shupei Liu, Yunlei Zhou, Jian Zhou, Hao Tang, Fei Gao, Decheng Zhao, Jinghui Ren, Yutong Wu, Zhoulu Wang, Yang Luo, Xiang Liu, Yi Zhang

**Affiliations:** School of Energy Sciences and Engineering, Nanjing Tech University, Nanjing 211816, Jiangsu Province, China; School of Mechanical Science and Engineering, Huazhong University of Science and Technology, Wuhan 430074, China; Empa, Swiss Federal Laboratories for Materials Science and Technology, ETH Domain, Dübendorf 8600, Switzerland

**Keywords:** energy storage device, flexible, preparation, Ti_3_C_2_T_*x*_MXenes

## Abstract

Over the past decade, two-dimensional (2D) Ti_3_C_2_T_*x*_ MXenes demonstrated attractive characteristics such as high electrical conductivity, tunable layered structure, controllable interfacial chemical composition, high optical transparency, and excellent electromagnetic wave absorption, enabling Ti_3_C_2_T_*x*_ MXenes as promising electrode materials in energy storage devices. Among these devices, flexible energy storage devices have attracted wide attention and developed rapidly due to the synchronously excellent electrochemical and mechanical properties. This review summarizes the recent progress of Ti_3_C_2_T_*x*_ MXenes pertaining to novel material preparation and promising applications in energy storage and conversion including batteries, supercapacitors, solar cells, and solar steam generation. This work aims to provide an in-depth and reasonable understanding of the relationship between the unique nanostructure/chemical composition of Ti_3_C_2_T_*x*_ MXenes and competitive electrochemical properties, which will facilitate the development of 2D Ti_3_C_2_T_*x*_ MXenes for practical energy storage and solar energy conversion devices.

## 1 Introduction

The two-dimensional material was proposed with the successful separation of graphene [1], which is prominently characterized by single atomic layer thickness, high carrier mobility, linear performance spectra, and high strength [2]. Subsequently, some other two-dimensional materials have been separated, such as boron nitride (BN), molybdenum disulfide (MoS_2_) and TMDCs [3, 4], MXene, etc. Two-dimensional materials are confined to the two-dimensional plane due to their carrier migration and heat diffusion, making the material exhibit many peculiar properties [5–10]. Such as the adjustable characteristics of the band gap, the controllability of the degree of spin freedom, and the special properties of the crystal structure lead to the anisotropy of different electrical properties or optical properties [9]. Compared to one-dimensional materials [11, 12], the above advantages make two-dimensional materials have broad application prospects in energy storage [13–17], catalysis [18, 19], optoelectronics [20, 21], flexible devices [22] and other fields. Ti_3_C_2_T_*x*_ MXenes are two-dimensional carbide materials with layered stacking structure similar to graphene [23]. In 2011, Ti_3_C_2_T_*x*_ MXenes were first reported by Gogotsi [24]. This work opens the door to the preparation and application of 2D MXenes. Then more and more researches have focused on the synthesis, properties, and applications of Ti_3_C_2_T_*x*_ MXenes [25]. The general formula of MXenes is M_*n*+1_X_*n*_T_*x*_, where M is transition metal, such as Ti, Mo, Nb, V, Cr, Zr, Ta, etc., X is carbon, nitrogen (*n* = 1–4), and T is the surface-functionalized groups. So far, more than thirty kinds MXenes have been reported, such as Mo_2_C [26, 27], V_2_C [28], Nb_2_C [29], Ti_4_N_3_ [30], TiNbC [31], etc. More than 100 kinds MXenes are predicted to exist. MXenes are usually prepared by the selective etching of layers from their MAX layered counterparts. The first reported Ti_3_C_2_T_*x*_ MXenes were prepared by etching Ti_3_AlC_2_ using hydrofluoric acid as an etchant by Gogotsi et al. etchants that have been reported include hydrofluoric acid (HF), HCl/LiF, HCl and fluoride salts, ammonium bifluoride (NH_4_HF_2_) or ammonium fluoride (NH_4_F), etc. During the etching process, functionalized groups (e.g., –F, –O, –OH) adsorb on the surface due to the interaction. Therefore, the surface property of Ti_3_C_2_T_*x*_ MXenes can be controlled by adjusting the synthesis conditions.

MXenes exhibit high electrical conductivity (up to 20,000 S/cm) [32], high stability, superior mechanical properties, and tunable layered structure. Therefore, MXenes have attracted increasing interest and become the focus of researchers. There are wide potential applications in batteries, supercapacitors, solar cells, and solar steam generation, electromagnetic interference (EMI) shielding materials [33–40]. In this review, we will present the recent advances in the preparation of MXenes-based flexible materials, MXenes-based energy storage and conversion applications including lithium-ion batteries, lithium-sulfur batteries, sodium-ion batteries, supercapacitors, solar cells, and solar steam generation. Finally, the development of MXenes-based flexible materials and MXenes-based energy storage and conversion applications are summarized and prospected.

## 2 Preparation of MXenes-based flexible materials

Nowadays, MXenes have been widely reported due to their excellent conductivity, superior flexibility, large surface area, and large tensile and compressive strengths, which enable them with wide applications in secondary batteries, supercapacitors, organic electronics, sensors, photocatalysis, etc. In particular, the flexibility of MXenes could enable them to be used in wearable electronic devices. Focusing on the technical issues of large-scale production and process synthesis, the preparation and application of MXenes-based flexible materials has become a potential development trend.

### 2.1 Coating methods

**Figure 1:**
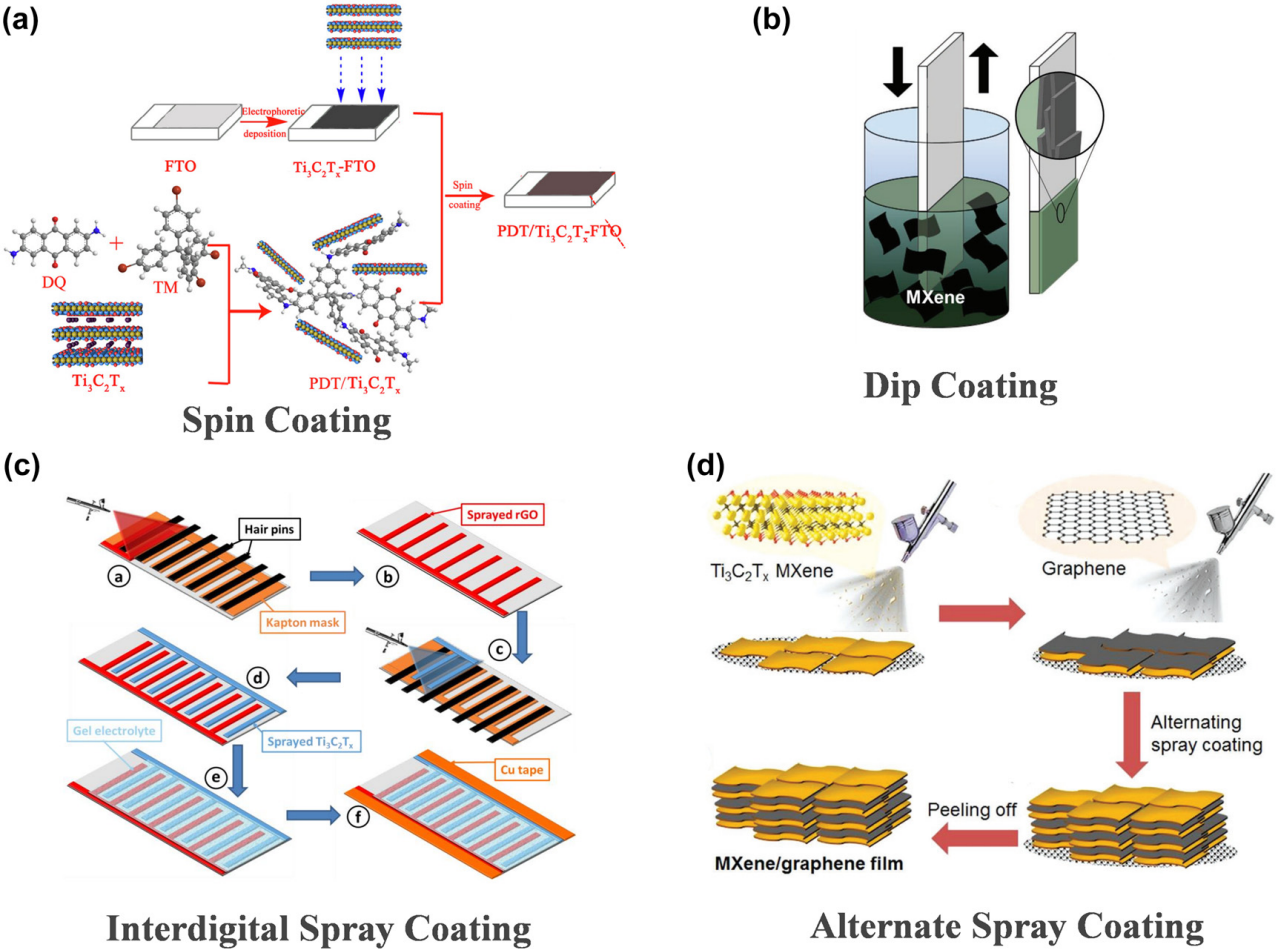
The methods of coating. (a) Schematic illustration for the fabrication of PDT/Ti_3_C_2_T_*x*_ film electrode. Reproduced with permission [41]. Copyright 2019, Elsevier B.V. (b) Schematic image of optically transparent Ti_3_C_2_ MXene film prepared by dip coating. Reproduced with permission [43]. Copyright 2018, WILEY-VCH. (c) Fabrication process of the asymmetric interdigitated MSC. Reproduced with permission [46]. Copyright 2017, WILEY-VCH. (d) Schematic showing the manufacturing of free-standing and flexible 2D MXene/graphene heterostructured films by a spray-assisted LbL process. Reproduced with permission [47]. Copyright 2019, WILEY-VCH.

Coating is a common method to prepare flexible materials, where the thickness can be tuned by controlling the material content in the solution. Various coating methods have been used to assemble flexible MXene films, including spin coating, spray coating, and dip coating. Spin coating typically uses a spin coater to convert preformed MXene dispersions into thin films. Wu et al. [41] reported the combination of a novel dispersed chain PDT and layered Ti_3_C_2_T_*x*_ MXenes by spin coating to form a free-standing hybrid film, which could enhance the flexible cycling performance (Figure 1(a)). As shown in Figure 1(b), dip coating usually involves dipping the substrate into the MXenes solution, and then removing the substrate and drying it in the air to obtain a flexible film [42, 43]. In spray coating, a spray gun is always used to spray the MXenes solution onto the substrate. Typically, the spray gun and heat gun are turned on to dry the coating immediately at the same time [44–47]. Cedric et al. reported the fabrication of MXenes-based asymmetric interdigitated micro-supercapacitors by spray coating MXenes solution on flexible PET substrates. The composite exhibited high flexibility during mechanical bending (Figure 1(c)). Zhao et al. reported alternately stacking MXenes and reduced graphene oxide (rGO) nanosheets by spray-assisted layer-by-layer assemble method (Figure 1(d)). Free-standing films with excellent flexibility can be obtained.

### 2.2 Self-assembly methods

**Figure 2:**
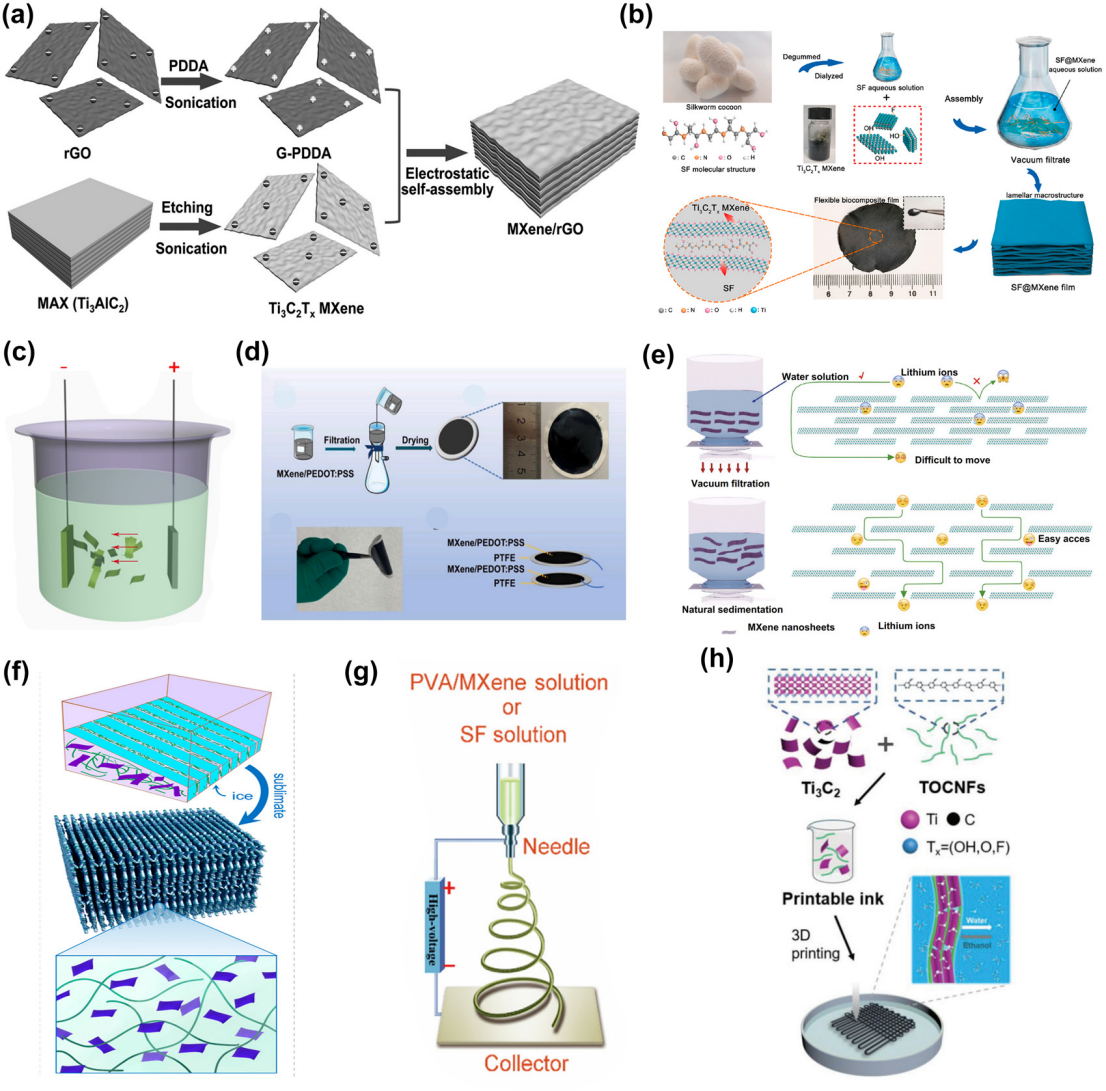
Some preparation methods for flexible materials. (a) Schematic illustration for the synthesis of the MXene/rGO hybrids. Reproduced with permission [48]. Copyright 2017, WILEY-VCH. (b) Illustration of preparation of the SF@MXene biocomposite film and flexible pressure sensor. Reproduced with permission [49]. Copyright 2020, Elsevier Ltd. (c) Schematic diagram of electrophoretic deposition apparatus. Reproduced with permission [50]. Copyright 2017, Elsevier B.V. (d) Illustration of the preparation of MPP film and TENG-based tactile sensor. Reproduced with permission [51]. Copyright 2021, Elsevier Ltd. (e) Comparison of improved ionic accessibility of the naturally sedimented MXene films and the conventional vacuum-filtered MXene film. Reproduced with permission [42]. Copyright 2020, the Author(s). (f) Freezing process of the mixed CNF/MXene precursor dispersion and corresponding freeze-dried lamellar porous scaffolds with large-scale aligned lamellar pores/cell walls. Reproduced with permission [52]. Copyright 2021, American Chemical Society. (g) Schematic diagram of electrospinning preparation; Reproduced with permission [53]. Copyright 2019, Elsevier Ltd. (h) Schematic illustration of the fabrication of smart TOCNFs/Ti_3_C_2_ fibers and textiles. Reproduced with permission [54]. Copyright 2019, WILEY-VCH.

Self-assembly methods include electrostatic self-assembly, electrophoretic deposition, vacuum-assisted filtration, etc. Yan et al. [48] proposed a simple method to prepare flexible films by electrostatic self-assembly between positive rGO and negative Ti_3_C_2_T_*x*_ MXenes nanosheets modified with poly(diallyldimethylammonium chloride). The film can facilitate rapid diffusion and transport of ions, as shown in Figure 2(a). Wang et al. [49] successfully fabricated SF@MXenes composite films by using flexible silk fibroin (SF) as a bridging agent to assemble pristine Ti_3_C_2_T_*x*_ MXenes nanosheets into a continuous layered macrostructure (Figure 2(b)). The electrophoretic deposition involves immersing electrodes in an MXenes solution to deposit MXenes on the electrodes at a constant voltage [55]. Furthermore, Xu et al. [50] fabricated a binder-free MXenes-based film by a modified electrophoretic deposition method using organic colloids containing a small amount of Ti_3_C_2_T_*x*_ MXenes nanoflakes (Figure 2(c)). Zhu et al. [34] prepared uniform I-Ti_3_C_2_T_*x*_ particle films by electrophoretic deposition. Due to the relatively weak interaction between PPy during polymerization, PPy was subsequently successfully intercalated into I-Ti_3_C_2_T_*x*_ particles via electrochemical polymerization to form a free-standing and conductive film. Zhang et al. [51] prepared integrated MXene-PEDOT: PSS/PTFE (MXene-poly(3,4-ethylenedioxythiophene): Poly(styrene sulfonate)/polytetrafluoroethylene) (MPP) films as electrodes by vacuum-assisted filtration method (Figure 2(d)). Recently, Sun et al. proposed a simple and effective strategy using natural precipitation to prepare free-standing flexible Ti_3_C_2_T_*x*_ MXenes films [42]. Compared with conventional vacuum-filtered MXene films, the natural precipitation films exhibited superior flexibility, and expanded interlayer distance, facilitating ion accessibility and ion transport, as shown in Figure 2(e).

### 2.3 Cross-linking methods

Freeze-drying techniques are commonly used to achieve cross-linking solidification of MXenes with other materials [56]. Gelation is a feasible method to assemble nanomaterials into different macroscopic structures. However, MXene nanosheets cannot form gels alone, so a suitable carrier must be selected. As shown in Figure 2(f), Zeng et al. [52] reported the preparation of light-weight layered porous biopolymer gas by embedding Ti_3_C_2_T_*x*_ MXenes into cellulose nanofibers (CNFs) via a bidirectional freeze-drying method. Feng et al. developed a conductive muscle-inspired ordered oriented MXenes hydrogel [57]. The conductive Ti_3_C_2_T_*x*_ MXenes nanosheets, biocompatible polymers (polyvinyl alcohol, PVA) and ZnSO_4_ solution were mixed. Then a hydrogel with an ordered internal orientation structure was prepared by repeated freezing and thawing process. The conductive hydrogel was used as a wearable flexible sensor in comprehensive human motion biomonitoring.

### 2.4 Spinning methods

Spinning is one of the simple, versatile, and convenient technologies for the production of nanofibers, commonly used in the production of one-dimensional fiber materials for polymers, metals, ceramics, and composites. In general, spinning methods mainly include electrostatic spinning and wet spinning. Mayerberger et al. [58] reported a nanofiber film prepared by curing MXenes with polyvinyl alcohol (PVA), polyacrylic acid (PAA), etc. Jiang et al. [53] innovatively integrated a highly electronegative and conductive MXenes nanosheet with polyvinyl alcohol (PVA) to electrospinning nanofiber films. A flexible all-electrospinning triboelectric nanogenerator (TENG) was fabricated (Figure 2(g)). Shao et al. successfully prepared polyester/Ti_3_C_2_T_*x*_ nanofiber-based yarns by electrospinning and applied them to supercapacitors [59]. Wet spinning is the preparation of MXene fibers using custom wet spinning equipment, such as MXene and polyurethane (PU) blending to produce MXene/PU fiber or wet spinning to obtain MXene fiber [60, 61].

### 2.5 3D printing

The 3D printing methods build 3D materials with complex geometries by joining or curing prefabricated ink materials. Direct ink writing (DIW) [62] and stereolithography [63] have been successfully used to produce 3D structured MXenes. The direct ink writing process is shown in Figure 2(h), which involves the extrusion and curing of high-viscosity MXenes ink. Stereolithography uses photopolymers for photocuring. MXenes nanosheets act as light retarders to improve printing quality by suppressing light scattering during printing and introducing excellent light-to-heat conversion properties. Cao et al. [54] inspired by natural materials, for the first time, flexible smart fibers and textiles are fabricated using a 3D printing process with hybrid inks of TEMPO (2,2,6,6‐tetramethylpiperidine‐1‐oxylradi‐cal)‐mediated oxidized cellulose nanofibrils (TOCNFs) and Ti_3_C_2_T_*x*_ MXenes. The hybrid inks display good rheological properties, which allow them to achieve accurate structures and to be rapidly printed. TOCNFs/Ti_3_C_2_T_*x*_ hybrid inks self‐assemble to fibers with an aligned structure in ethanol. TOCNFs/Ti_3_C_2_T_*x*_ textiles with electromechanical performance can be processed into sensitive strain sensors. For reference, we summarize the common preparation methods of Ti_3_C_2_T_*x*_ MXenes-based flexible materials in Table 1, and the corresponding flexible materials are also listed.

**Table 1:** Preparation methods of MXenes-based flexible materials.

Methods		Materials	Ref.
Coating	Spin coating	PDT/Ti_3_C_2_T_*x*_ MXene	[41]
	Spray coating	Ti_3_C_2_T_*x*_ MXene	[46]
	Dip coating	Ti_3_C_2_T_*x*_ MXene	[43]
Self-assembly	Electrostatic self-assembly	MXene/rGO	[48]
	Self-assembly	SF@MXene	[49]
	Electrophoretic deposition	Ti_3_C_2_T_*x*_ MXene	[50]
	Vacuum-assisted filtration	MPP films	[51]
	Natural precipitation	Ti_3_C_2_T_*x*_ MXene	[42]
Cross-linking	Freeze-drying	Ti_3_C_2_T_*x*_ MXene/CNF	[52]
Spinning	Electrospinning	Ti_3_C_2_T_*x*_ MXene	[58]
	Wet spinning	MXene/PU	[60]
3D printing	Direct ink writing	Ti_3_C_2_T_*x*_ MXene	[62]
	Stereolithography	MXene/SA/PAM	[63]

## 3 Application of Ti_3_C_2_T_*x*_ MXenes in energy storage and conversion

2D materials have attracted extensive attention due to their controllable interfacial chemistry [64], high electronic conductivity, high optical transparency [65, 66], and tunable layered structure, which make 2D Ti_3_C_2_T_*x*_ MXenes a promising electrode material in energy storage devices [15, 67, 68]. In addition, it is widely used in various fields such as lithium-ion batteries [69], lithium–sulfur batteries [70], sodium-ion batteries [71, 72], supercapacitors [73], solar cells [74], and solar steam generation [75], etc. [76, 77].

### 3.1 Batteries

#### 3.1.1 Lithium-ion batteries

**Figure 3:**
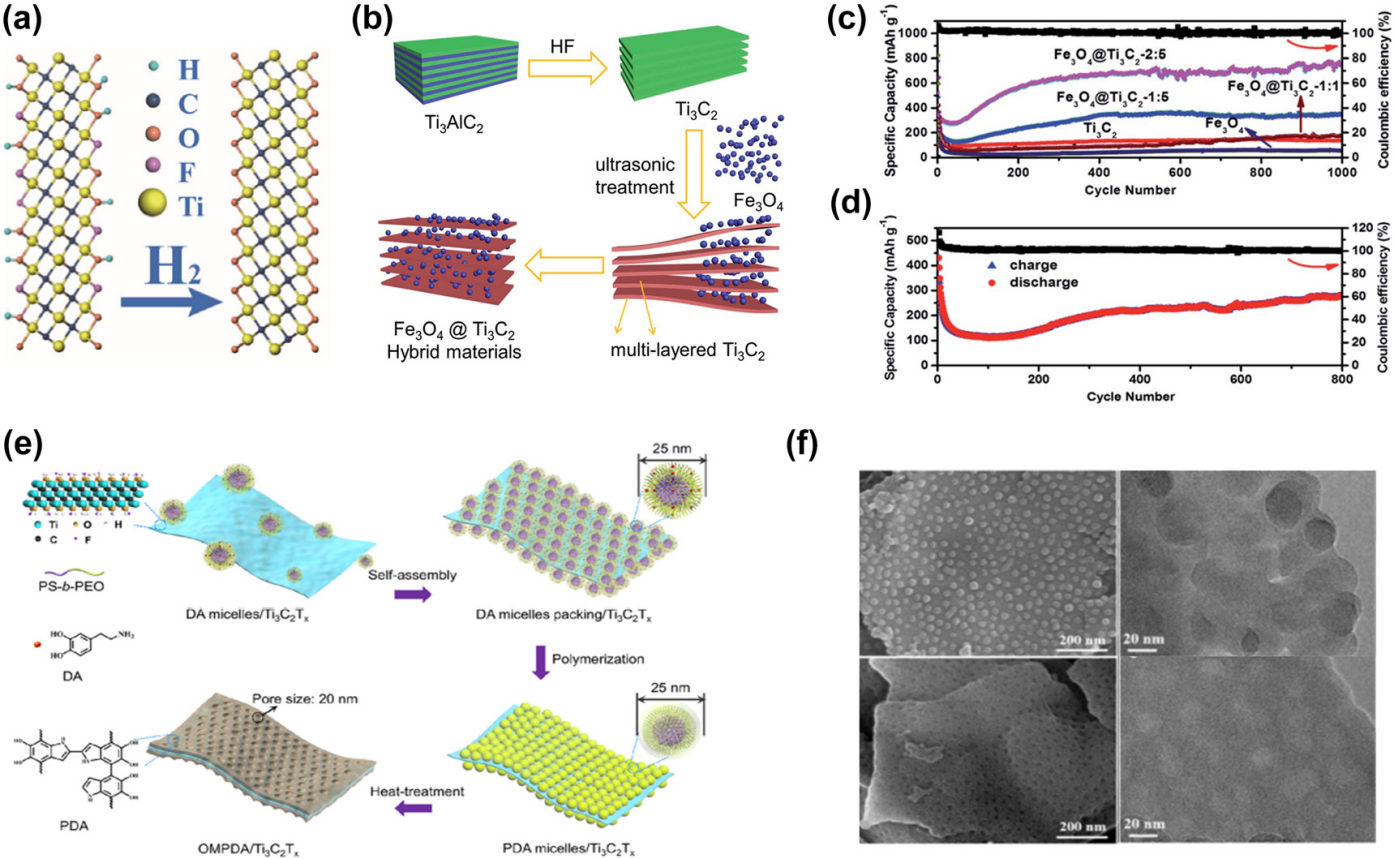
The structural models of Ti_3_C_2_T_x_ composites. (a) The structural models of MX and MX-H_2_. Reproduced with permission [80]. Copyright 2018, Elsevier B.V. (b) Schematic showing the preparation process of Fe_3_O_4_@Ti_3_C_2_ hybrids. (c) Long cycling performance of Ti_3_C_2_, Fe_3_O_4_ and Fe_3_O_4_@Ti_3_C_2_ hybrid electrodes at 1C. (d) long cycling performance of Fe_3_O_4_@Ti_3_C_2_-2:5 at 5C. Reproduced with permission [81]. Copyright 2018, The Royal Society of Chemistry. (e) Schematic drawing depicting the preparation steps of OMPDA/Ti_3_C_2_T_*x*_ composite. (f) SEM and TEM images of PDA mi/Ti_3_C_2_T_*x*_. SEM and TEM images of OMPDA/Ti_3_C_2_T_*x*_. Reproduced with permission [82]. Copyright 2020, American Chemical Society.

In recent decades, Li-ion batteries have received extensive attention due to their high specific energy, high cycle stability, and no memory effect, and have been widely used in portable electronics and electric vehicles. The performance of Li-ion batteries depends largely on the electrode materials. Among various developed electrode materials, 2D materials have high surface-to-volume ratios, fast ion transport channels, and short-distance interaction, thus 2D materials play a key role in LIBs. As a novel class of 2D materials, Ti_3_C_2_T_*x*_ MXenes show great potential in next-generation LIBs [78, 79]. Lu et al. [80] prepared F-Ti_3_C_2_T_*x*_ MXenes (Figure 3(a)). The electrochemical test showed that changing the MXenes surface functional group concentration can improve the kinetics of lithium-ion transport between the electrolyte and electrodes.

An effective strategy to realize the potential of MXenes for high-performance lithium-ion batteries is to combine MXenes with other anode materials, such as transition metal oxides. Composites based on Ti_3_C_2_T_*x*_ and Fe_3_O_4_ exhibit high capacity [81] (Figure 3(b)). In the structure, Fe_3_O_4_ nanoparticles are uniformly dispersed on the surface of MXenes. Due to the metallic conductivity and good lithium storage ability of the composites, the Fe_3_O_4_@Ti_3_C_2_T_*x*_ was prepared and showed better electrochemical performance compared with pure Fe_3_O_4_ or Ti_3_C_2_T_*x*_. This nanohybrid exhibits a capacity of 747.4 mAh g^−1^ after 1000 cycles at 1C (Figure 3(c)). At 5C, the capacity after 800 cycles is 278.3 mAh g^−1^ (Figure 3(d)). The nontoxicity of SnO_2_ makes it another attractive anode material [83]. Liu et al. proposed a method to realize electrostatic self-assembly of SnO_2_ quantum dots (QDs) on Ti_3_C_2_T_*x*_-MXenes sheets. The SnO_2_ QDs/MXenes exhibit excellent lithium storage performance (887.4 mAh g^−1^ at 50 mA g^−1^), stable cycling performance (659.8 mAh g^−1^ at 100 mA g^−1^ with capacity retention of 91% after 100 cycles), and excellent rate capability (364 mAh g^−1^ at 3 A g^−1^). Ti_3_C_2_T_*x*_ flakes may reduce ion transport distance. A sandwich-structured ordered mesoporous polydopamine OMPDA/Ti_3_C_2_T_*x*_ composite was developed [82] (Figure 3(e)). The OMPDA layer has vertically oriented and accessible nanopores (∼20 nm), which provides continuous channels for ion diffusion. The Ti_3_C_2_T_*x*_ layers guarantee a fast electron transfer path. Compared with common polymer-based electrodes, the lithium storage performance of the as-prepared OMPDA/Ti_3_C_2_T_*x*_ composite is significantly enhanced. The OMPDA/Ti_3_C_2_T_*x*_ composite exhibits high reversible capacity, long cycling stability and good rate performance. *In situ* TEM analysis revealed that OMPDA in the composite retains the initial morphology during lithiation (Figure 3(f)). Furthermore, a stable and ultrathin solid-electrolyte interfacial layer is formed in the active material, which acts as an electrode protective film during cycling.

**Figure 4:**
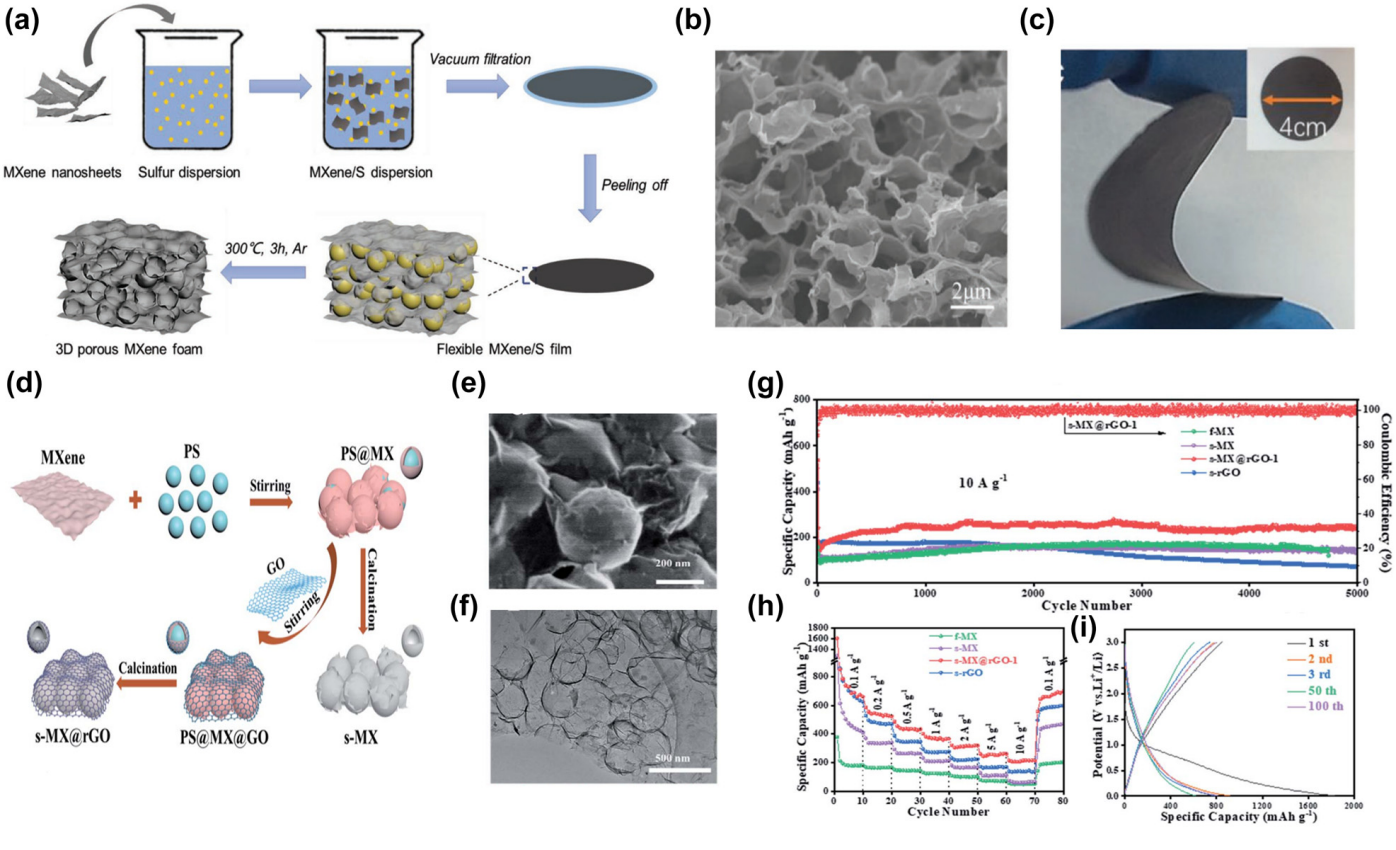
Preparation process, structure and electrochemical performance of flexible 3D MXene foam. (a) Schematic illustration of the preparation process of the freestanding and flexible 3D porous MXene foam. (b) SEM images and (c) digital photo of p-MXene-71. Reproduced with permission [84]. Copyright 2019, WILEY-VCH. (d) Schematic illustration of the fabrication process of the s-MX@rGO composite. (e,f) SEM and TEM images of s-MX@rGO-1. (g) long-term cycling properties at 10 A g^−1^ for 5000 cycles; (h) rate capability at various current densities (0.1 A g^−1^–10 A g^−1^). (i) Charge/discharge curves of the s-MX@rGO-72 electrode in different cycles at 100 mA g^−1^. Reproduced with permission [85]. Copyright 2021, The Royal Society of Chemistry.

Furthermore, the 3D porous electrode design can effectively achieve ion transport and increase the use of MXene nanosheets for improved lithium storage performance [86]. Zhao et al. [84] proposed the use of sulfur as a template to construct a 3D MXenes foam with a well-developed porous structure (Figure 4(a) and (b)). The capacity of this flexible 3D porous MXenes foam is 455.5 mAh g^−1^ at 50 mA g^−1^, 101 mAh g^−1^ at 18 A g^−1^, and 220 mAh g^−1^ at 1 A g^−1^ after 3500 cycles. By another approach, a uniform three-dimensional (3D) hollow MXenes nanosphere with an average diameter of <300 nm was synthesized by a template method [85] (positively charged polystyrene). In addition, reduced graphene oxide (rGO) was also introduced to wrap hollow MXenes nanospheres to form hybrid material MXenes@rGO nanospheres (Figure 4(d)). SEM and TEM images show that spheres with diameters of 250–300 nm are cross-linked with each other by MXenes and rGO, presenting a hollow structure (Figure 4(e) and (f)). Therefore, hollow MXenes@rGO nanospheres exhibit excellent rate capability (241.5 mAh g^−1^ after 5000 cycles at 10 A g^−1^) (Figure 4(g) and (h)). Figure 4(i) are the charge–discharge curves. It demonstrated that 3D MXenes@rGO nanospheres are promising anode materials for high-rate Li-ion batteries.

#### 3.1.2 Lithium–sulfur batteries

Lithium–sulfur (Li–S) batteries are considered to be one of the most competitive batteries in the next generation of secondary energy storage batteries due to their extremely high theoretical specific capacity (1675 mAh g^−1^) [87–89]. However, lithium-sulfur batteries still face some serious challenges, such as poor rate performance, and short cycle life by the “shuttle effect” [90, 91]. To solve the above-mentioned problems, various methods have been proposed [92, 93]. Using host materials with high electrical conductivity and surface area is one of the most common strategies to confine lithium polysulfides (LIPS) within cathodes. So far, cathode hosts such as porous carbon, hollow carbon spheres, graphene, TiO_2_ hollow spheres, COFs, MOFs, etc. have been reported [94–99].

**Figure 5:**
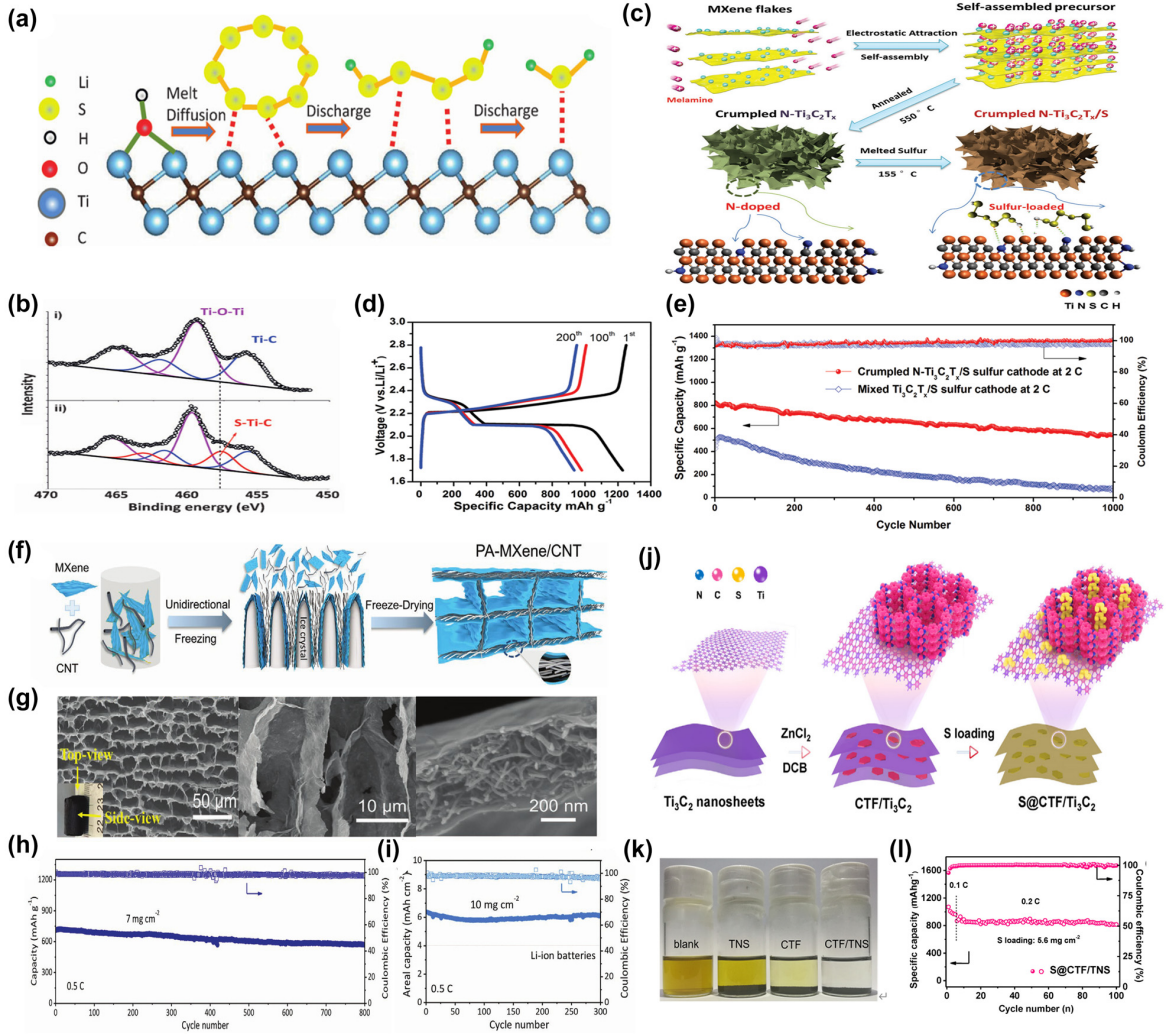
Preparation process, structure and electrochemical performance of S/MXene cathode for Li-S battery. (a) Replacement of the Ti-OH bond on the MXene surface with a S-Ti-C bond on heat treatment or by contact with polysulfides. (b) Ti 2p spectra of the (i) Ti_2_C sheets and (ii) 70 S/Ti_2_C composite. Reproduced with permission [100]. Copyright 2015, WILEY-VCH. (c) Illustration of synthesis of the crumpled N-Ti_3_C_2_T_*x*_/S composites. (d) Typical charge and discharge profiles of crumpled N-Ti_3_C_2_T_*x*_/S electrodes for the 1st, 100th, and 200th cycle at 0.2C. (e) Cycling performances of crumpled N-Ti_3_C_2_T_*x*_/S electrodes and mixed-Ti_3_C_2_T_*x*_/S electrodes at 2C for 1000 cycles (1C = 1673 mA g^−1^). Reproduced with permission [101]. Copyright 2018, WILEY-VCH. (f) The schematic of the assembly process of the PA-MXene/CNT aerogel by the unidirectional freeze-drying. (g) SEM images of PA-MXene/CNT. (h, i) Cycling performance of PA-MXene/CNT-50 electrode with sulfur loadings of 7 and 10 mg cm^−2^ at 0.5 C. Reproduced with permission [96]. Copyright 2021, Wiley-VCH GmbH. (j) Schematic illustration of the synthetic process of the 2D CTF/TNS heterostructures and the S@CTF/TNS composites. (k) Digital photos of Li_2_S_6_ adsorption. (l) Cycling performance of the S@CTF/TNS electrode with a high areal sulfur loading of 5.6 mg cm^−2^. Reproduced with permission [104]. Copyright 2020, Elsevier Ltd.

Ti_3_C_2_T_*x*_ MXenes is a promising sulfur host candidate due to its high electrical conductivity and unique two-dimensional structure similar to graphene. Liang firstly used Ti_3_C_2_T_*x*_ MXenes as a sulfur host material [100]. Taking advantage of the highly conductive and highly reactive 2D surfaces possessed by MXenes, intermediate polysulfides are chemically bonded via metal-sulfur interactions (Figure 5(a)). X-ray photoelectron spectroscopy (XPS) analysis showed the formation of strong Ti-S interactions between titanium atoms and polysulfides (Figure 5(b)). The S/Ti_3_C_2_T_*x*_ composite with 70% sulfur demonstrated excellent cycling stability with a discharge capacity of 723 mAh g^−1^ after 650 cycles, corresponding to a decay rate of 0.05% per cycle. Bao et al. [101] synthesized N-Ti_3_C_2_T_*x*_ MXenes nanosheets with strong physical and chemical dual adsorption of polysulfides by a novel one-step method (Figure 5(c)). The N-Ti_3_C_2_T_*x*_ nanosheets were prepared by thermal annealing of positively charged melamine and negatively charged metallic Ti_3_C_2_T_*x*_ flakes. N-Ti_3_C_2_T_*x*_/S composites exhibit outstanding electrochemical performance, including high reversible capacity (1144 mAh g^−1^ at 0.2 C) and long cycling stability (610 mAh g^−1^ at 2C after 1000 cycles) (Figure 5(d) and (e)) [102, 103].

In order to meet the growing demand for commercialization, achieving long-cycle stability under high sulfur loading is a basic requirement for the practical use of lithium-sulfur batteries. Zhang [96] proposed a proof-of-concept host for a free-standing MXenes/CNT composite aerogel consisting of interconnected and parallel-arranged sandwiches (Figure 5(f) and (g)), which can achieve ultra-stable cycling at high sulfur content. The S/MXenes/CNT composite exhibits a high capacity of 712 mAh g^−1^ with a sulfur loading of 7 mg cm^−2^ and exhibits excellent cycling stability with capacity decay per cycle of 0.025% after 800 cycles at 0.5 C. With sulfur loading of 10 mg cm^−2^, a high areal capacity above 6 mAh cm^−2^ was obtained after 300 cycles (Figure 5(h) and (i)).

Recently, *in situ* growth of COFs on various carbon-based materials has been widely reported [105, 106]. Yang’s group [104] synthesized a heterostructure by growing a layered covalent triazine framework (CTF) on Ti_3_C_2_ MXenes nanosheets (TNS) (Figure 5(j)). The lithiophilic N sites in CTF and the thiophilic Ti sites in TNS allow the polysulfides to be chemically anchored and the shuttle effect is effectively suppressed (Figure 5(k)). With a high sulfur loading of 76%, the S@CTF/TNS cathode exhibits high reversible specific capacity (1441 mAh g^−1^ at 0.2C), excellent rate performance, and cycling stability. When the areal loading of the active material was 5.6 mg cm^−2^, a capacity retention rate of 94% was still obtained after 100 cycles (Figure 5(l)).

Furthermore, Wei et al. [107] obtained a flexible, free-standing MXenes/COF. COF-LZU1 microspheres are distributed in the MXenes membrane framework. Lithophilic COF-LZU1 microspheres act as nucleation seeds, which can promote the uniform nucleation of lithium by homogenizing the Li^+^ flux and reducing the nucleation barrier. Under the regulation of COF-LZU1 seeds, the Coulombic efficiency of the MXenes/COF-LZU1 framework and the electrochemical stability are significantly enhanced. Lithium-sulfur batteries using modified lithium metal anodes and sulfide polyacrylonitrile (S@PAN) cathodes exhibit superior electrochemical properties. In addition, COF@MXenes heterostructures have also been applied in the modification of Li-S battery separators to improve their electrochemical performance [108].

#### 3.1.3 Other batteries

With the continuous consumption of lithium resources, there is an urgent need to develop new and resource-rich batteries. Rechargeable sodium, potassium, zinc, magnesium, and aluminum ion batteries have received extensive attention [109–113]. MXenes show great potential as electrode materials in these batteries. Using DFT methods, various ions (Li^+^, Na^+^, K^+^, and Mg^2+^) are embedded in a large number of M_2_C MXenes -based compounds (M = Sc, Ti, V, Cr, Zr, Nb, Mo, Hf, Ta) to have –F, –H, –O and –OH functionalized surfaces [114]. In terms of gravimetric capacity, a greater amount of Li^+^ or Mg^2+^ can be intercalated into an MXene than Na^+^ or K^+^, which is related to the size of the intercalating ion. Variation of the surface functional group and transition metal species can significantly affect the voltage and capacity of an MXene, with oxygen termination leading to the highest capacity, which shows that MXenes are promising candidates for rechargeable batteries.

**Figure 6:**
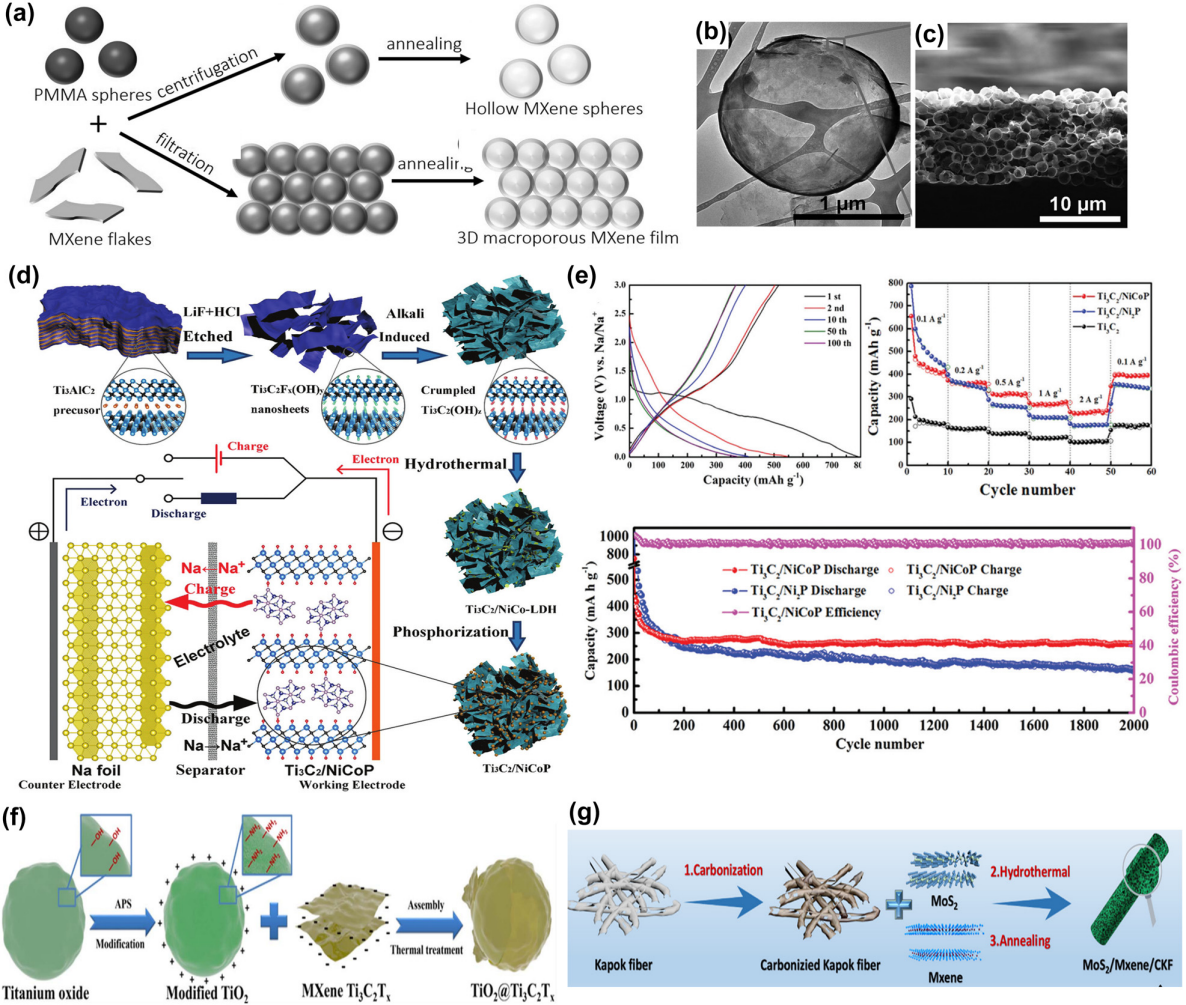
Preparation process, structure and electrochemical performance of MXene composites anode for SIBs. (a) Schematic showing the construction of hollow MXene spheres and 3D macroporous MXene frameworks. (b) A hollow Ti_3_C_2_T_*x*_ sphere. (c) Cross-sectional SEM images of the 3D macroporous Ti_3_C_2_T_*x*_ film. Reproduced with permission [118]. Copyright 2017, WILEY-VCH. (d) Schematic illustration of the synthesis process of the Ti_3_C_2_/NiCoP hybrid and schematic mechanism of half-cells. (e) Discharge–charge profiles of the Ti_3_C_2_/NiCoP electrode. Rate capability of the Ti_3_C_2_, Ti_3_C_2_/Ni_2_P, and Ti_3_C_2_/NiCoP electrodes. Long cycling performance of Ti_3_C_2_/Ni_2_P, and Ti_3_C_2_/NiCoP electrodes at a current density of 1.0 A g^−1^ after 2000 cycles. Reproduced with permission [122]. Copyright 2019, The Royal Society of Chemistry. (f) Schematic illustration of the synthetic process for TiO_2_@Ti_3_C_2_T_*x*_ material. Reproduced with permission from Reference [126]. Copyright 2018, Elsevier B.V. (g) Schematic diagram of the synthesis process for MoS_2_/Mxene/CKF electrode. Reproduced with permission [123]. Copyright 2021, Elsevier Inc.

Due to the scarcity and uneven distribution of lithium in the Earth’s crust, there is an urgent need to develop a new generation of energy storage devices. Rechargeable sodium-ion batteries are considered promising alternatives to LIBs due to their similar chemistry to lithium, and the abundance and low cost of metallic sodium. Compared with Li^+^ (0.76 Å), Na^+^ has a larger radius (1.06 Å) which results in slow intercalation kinetic mechanism. The difficult mass transport and storage during electrochemical processes result in poor electrochemical rate and cycling performance [114–116]. Therefore, the commercial graphite anode material may not be suitable for SIBs because it may not store enough sodium ions. Due to the open ion transport channels and large specific surface area of 2D layered materials, they can provide a large number of active sites for the storage of sodium ions. As a novel two-dimensional material, MXenes have been investigated to reveal the effect of interlayer expansion on the storage and diffusion of sodium by density functional theory [117]. Theoretical results indicate that the –F, –O, and –OH functionalized Ti_3_C_2_T_*x*_ MXenes have lower potential barriers. In addition, the increased interlayer distance enables stable adsorption of Na+, thus significantly increasing its theoretical capacity. However, MXenes nanosheets tend to stack together, limiting device performance. Zhao et al. [118] reported on processing Ti_3_C_2_T_*x*_ MXenes into 3D macroporous frameworks via poly(methyl methacrylate) (PMMA) spherical templates. The prepared 3D macroporous Ti_3_C_2_T_*x*_ films are independent, flexible and have high electrical conductivity. When it was used as an anode for sodium-ion storage, it exhibited high performance in terms of capacity, rate capability and cycling stability compared to multilayer MXenes and MXenes/carbon nanotube hybrid structures (Figure 6(a)–(c)).

To further enhance the structural stability and improve the slow electrochemical reaction kinetics, MXenes are combined with other high-capacity materials [119–121]. This approach prevents the repacking of Ti_3_C_2_T_*x*_ MXenes nanosheets, resulting in a large interlayer spacing and improving the sodium storage capacity. Ti_3_C_2_T_*x*_ MXenes/NiCoP 3D interconnected porous structures were successfully designed as anodes for high-performance SIBs [122]. The porous structures were formed through a solvothermal reaction and *in situ* phosphorization process (Figure 6(d)). The unique structure can effectively tolerate volume expansion and prevent aggregation and pulverization of NiCoP nanoparticles during Na^+^ insertion/extraction. The as-prepared Ti_3_C_2_T_*x*_ MXenes/NiCoP electrode exhibited large reversible capacity, high rate capacity, and excellent cycling performance (Figure 6(e)). Furthermore, some transition metal oxides, sulfides or selenides were introduced to enhance the ion storage capacity of 3D MXenes [123–125]. Guo et al. [126] reported a new strategy to synthesize TiO_2_@Ti_3_C_2_T_*x*_ MXenes/NiCoP composites as anode materials for NIBs (Figure 6(f)). The MXenes layers significantly enhance the electronic conductivity of the electrode and protect the structural integrity of the TiO_2_ spheres. Therefore, it contributes to the formation of a stable solid electrolyte interface. As a result, the hybrid electrode delivers a high reversible capacity of 116 mAh g^−1^ at 960 mA g^−1^ after 5000 cycles. And full cell achieves a capacity of 103.4 mAh g^−1^ at 960 mA g^−1^ and an excellent cycling performance (73.5% capacity retention after 1000 cycles).

Molybdenum disulfide (MoS_2_) with layered structure and high theoretical capacity is an anode material candidate for NIBs [123]. A hierarchical structure based on self-assembled MoS_2_ nanoflowers, MXenes, and hollow carbonized kapok fibers (CKF) was designed (Figure 6(g)). Nanomaterials can be connected to form heterojunctions, avoiding agglomeration. The loading of the heterostructure and the stress release of the CKF are coordinated to form a dual protection mechanism, which improves the electrical conductivity and structural stability of the hybrid material. Based on the above advantages, it has a high specific capacity and better rate capability than pure MoS_2_ or MXenes. Furthermore, besides exploring Ti_3_C_2_T_*x*_ MXenes as SIB anodes, other MXenes such as Nb_2_CT_*x*_, Ca_2_N, Sr_2_N, V_2_CT_*x*_, and Mo_2_C have also attracted great interest in sodium storage applications [27,127], [128], [129].

Potassium-ion batteries (KIBs) have attracted widespread attention due to their similar chemistry to lithium and sodium, natural abundance, and low cost of potassium sources. However, the huge volume expansion and sluggish kinetics caused by the large radius of K^+^ lead to the low capacity and poor cycle life. Therefore, researchers mainly focus on developing new layered materials. 2D layered materials have large specific surface areas and open 2D ion conduction pathways, which provide a number of active sites for the storage of potassium ions [76, 131, 132]. MXenes have attracted great interest as potential electrode materials. Here, several studies report the development of high-performance KIB anode materials based on MXenes. Lian et al. synthesized MXenes-based nanoribbons (a-Ti_3_C_2_T_*x*_) by treating pristine Ti_3_C_2_T_*x*_ MXenes with continuous shaking in aqueous KOH [133]. The as-prepared a-Ti_3_C_2_T_*x*_ exhibits an extended interlayer spacing of 12.5 Å, an oxygen-terminated surface, a narrow width of 6–22 nm, and a 3D porous interconnecting framework, which is beneficial for increased ion storage and fast ionization along with interlayer nanochannels diffusion. The obtained a-Ti_3_C_2_T_*x*_ anodes showed excellent Na^+^/K^+^ storage performance (high reversible capacities of 168 mA h g^−1^ at 20 mA g^−1^, 84 mA h g^−1^ at 200 mA g^−1^ for SIBs, 136 mA h g^−1^ at 20 mA g^−1^ and 78 mA h g^−1^ at 200 mA g^−1^ for PIB). Huang et al. [134] proposed to grow MoSe_2_ nanosheets on highly conductive MXenes flakes by hydrothermal method, and then coat the MoSe_2_/MXenes hybrid nanosheets with a polydopamine (PDA)-derived carbon layer (denoted as MoSe_2_/MXenes@C). MoSe_2_ nanosheets are vertically anchored on the MXenes substrate to form a layered 2D nanosheet structure, which can effectively alleviate the aggregation and stacking of MoSe_2_ nanosheets and MXenes. As an anode material for PIB, the obtained MoSe_2_/MXenes@C can achieve a high reversible capacity of 355 mAh g^−1^ after 100 cycles at 200 mA g^−1^ and a high reversible capacity of 183 mAh g^−1^ at 10.0 A g^−1^.

Considering the low structural stability and poor electrochemical redox kinetics caused by the large size of K^+^ (1.38 Å), a novel PDDA-NPCN/Ti_3_C_2_T_*x*_ hybrid was designed as an anode for PIBs [135]. The coupled PDDA-NPCN/Ti_3_C_2_T_*x*_ hybrid with a stacked structure and large specific surface area can ensure tight contact between Ti_3_C_2_T_*x*_ and NPCN, resulting in more accessible active sites. A high reversible capacity of 358.4 mAh g^−1^ and long-cycle stability of 252.2 mAh g^−1^ after 300 cycles at 0.1 A g^−1^ with only 0.03% loss per cycle after 2000 cycles at 1.0 A g^−1^ were obtained. In order to improve the potassium storage cycling ability, graphite nanosheet (GNF)/MXenes (GNFM) electrodes have been rationally designed [136]. The unique structures exhibit high structural stability and flexibility and achieve excellent potassium storage capacity, cyclability, and rate performance. Another class of antimony-based materials has been used as PIB electrode material due to the advantage of high capacity [137]. Wang et al. [137] synthesized Sb_2_S_3_ nanoflowers on the surface of Ti_3_C_2_T_*x*_ MXenes flakes by a solvothermal reaction and calcination method. Rational structural design can not only improve the charge transfer kinetics but also buffer the volume expansion during K^+^ intercalation and deintercalation.

**Figure 7:**
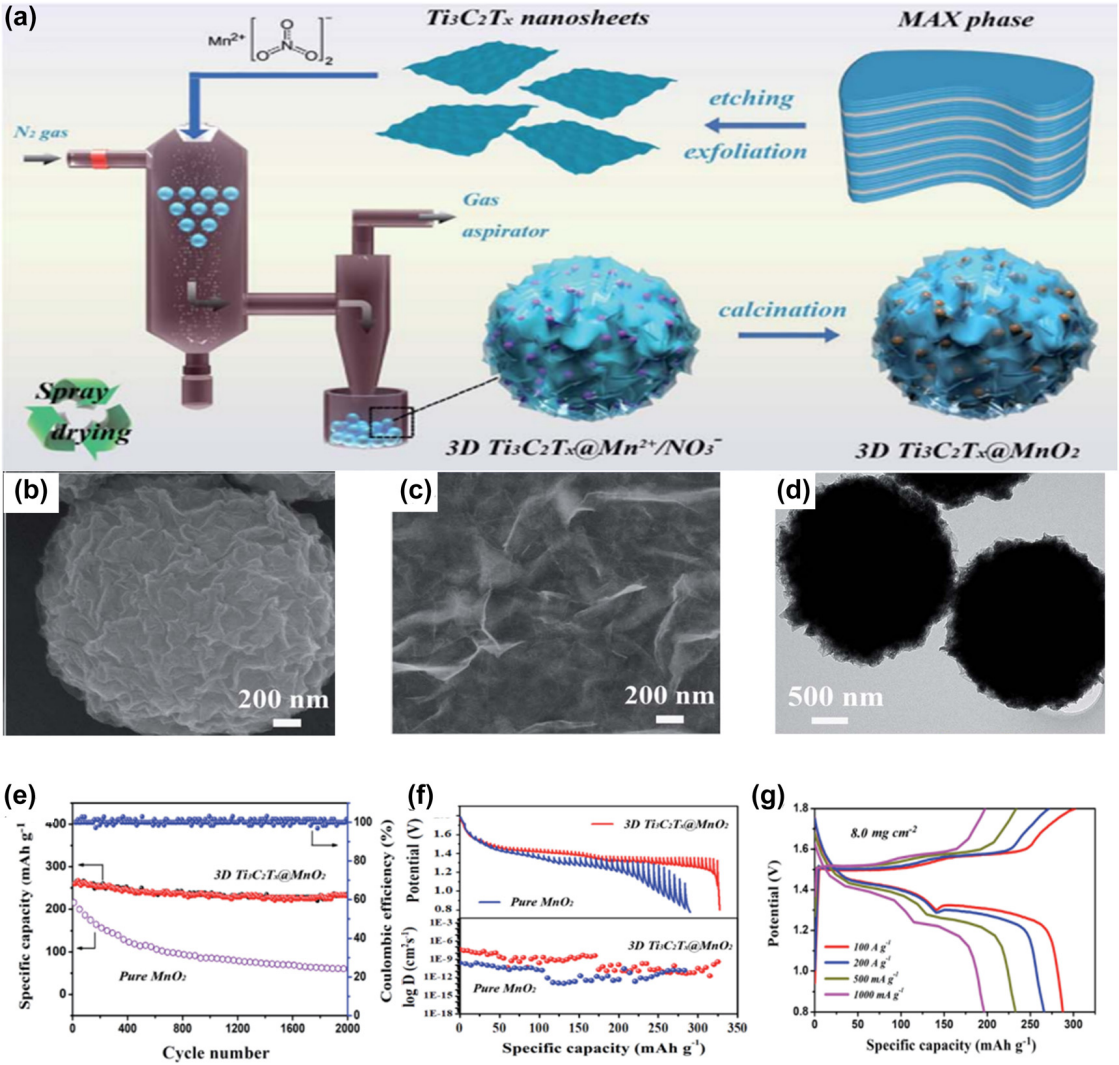
The structure and electrochemical performance of MXene composites anode for SIBs. (a) Schematic illustration of the synthesis process of 3D Ti_3_C_2_T_*x*_@MnO_2_ microflowers. (b) SEM images of 3D Ti_3_C_2_T_*x*_@MnO_2_ microflowers. (c) Ultrathin Ti_3_C_2_T_*x*_ nanosheets after ultrasonic exfoliation. (d) TEM images of 3D Ti_3_C_2_T_*x*_@MnO_2_ microflowers. (e) Long-term cycling stability (current density of 500 mA g^−1^) evaluated using the coulombic efficiency of 3D Ti_3_C_2_T_*x*_@MnO_2_ microflowers. (f) GITT curves of pure MnO_2_ and 3D Ti_3_C_2_T_*x*_@MnO_2_ microflower cathodes. (g) GCD profiles of a 3D Ti_3_C_2_T_*x*_@MnO_2_ microflower cathode with a high mass loading of 8.0 mg cm^2^. Reproduced with permission [130]. Copyright 2020, The Royal Society of Chemistry.

Aqueous zinc-ion batteries (ZIBs) have attracted attention due to their non-toxic, non-flammable, and low cost [138–141]. By introducing the Ti_3_C_2_T_*x*_ MXene electrode for ZIB, high energy density and excellent power transfer can be achieved. Luo et al. [142] recorded MnOx@Ti_3_C_2_T_*x*_ based on accordion-shaped Ti_3_C_2_T_*x*_ nanosheets and MnOx. As the current increasing from 0.1 to 10 A g^−1^, 50% initial capacity of MnOx@Ti_3_C_2_T_*x*_ was maintained while the power and energy densities were 25 W L^−1^ and 80 Wh L^−1^. Li et al. [143] reported a vertically aligned Sn^4+^ pre-intercalated MXenes sphere. After pre-intercalation, the interlayer spacing is effectively increased (from 1.15 nm to 1.27 nm), which can shorten the ion diffusion path and enhance ion transport. The capacity is 138 mAh g^−1^ at 0.1 A g^−1^ and 92 mAh g^−1^ at 5 A g^−1^. Shi et al. [144] synthesized 3D ZMO@Ti_3_C_2_T_*x*_, in which highly conductive Ti_3_C_2_T_*x*_ could suppress the irreversible structural degradation and side reactions of spinel ZMO. Therefore, the ZMO@Ti_3_C_2_T_*x*_ composite cathode has a high reversible specific capacity, excellent rate performance and long-term cycling stability (the capacity retention rate after 5000 cycles is about 92.4%). Li et al. [145] demonstrated a zinc mixed-ion battery (ZHIB) by using MXenes as the cathode, the capacity was exceptionally enhanced within 18,000 cycles, which was in agreement with all reported in Batteries that start to degrade within hundreds of cycles are very different. The main mechanism was identified as an unexpected phase transition during MXenes delamination and cycling. Both the original cathode and the second derivative have an effect on the capacitance, resulting in an unusual capacitance enhancement. As a result, a specific capacity of 508 mAh g^−1^ and a high energy density of 386.2 Wh kg^−1^ are achieved. To further develop inexpensive and stable electrode material, Shi et al. [130] developed a novel 3D high-density MXenes-MnO_2_ composite cathode material by gas-phase spray drying strategy, in which MnO_2_ nanoparticles were encapsulated in crumpled corrugated MXenes nanoparticles (Figure 7(a)–(d)). GITT was implemented to evaluate the ionic diffusivity which reached the 10^−8^ to 10^−10^ cm^2^ s^−1^ (Figure 7(f)), much higher than that of the pure MnO_2_ cathode. 3D MXenes@MnO_2_, used as a ZIB cathode, exhibited a high reversible specific capacity of 301.2 mAh g^−1^ over 2000 cycles, remarkable rate capability and excellent cycling stability. When the mass loading was increased to 8.0 mg cm^2^, the specific capacity was 287.6 mAh g^−1^ (Figure 7(e) and (g)). Rechargeable zinc-based batteries are a promising power source due to their low cost and safety. However, zinc dendrites and side reactions limit the practical application of zinc metal anodes. Tian et al. [146] designed multifunctional uniform antimony (Sb) nanoarrays and grown them on Ti_3_C_2_T_*x*_ MXenes paper. The optimized free-standing MXenes@Sb electrode outputs a capacity of 299.6 mAh g^−1^ over 200 cycles at 50 mA g^−1^ and maintains 148.43 mAh g^−1^ after 1000 cycles at 500 mA g^−1^. It is proved that zinc can be alloyed with antimony. This study demonstrates the feasibility of antimony as an alloyed zinc storage anode, providing an effective way to suppress zinc dendrites.

### 3.2 Supercapacitors

#### 3.2.1 Ti_3_C_2_T_*x*_/carbon composites

Compared with conventional batteries, supercapacitors (SCs) have attracted much attention due to their high power density. There are two types of supercapacitors: electric double-layer capacitors (EDLC) and pseudocapacitors. EDLC storage energy by adsorbing ions and forming an electric double layer at the electrode-electrolyte interface [147]. The capacity of EDLC is related to the surface area of the electrode. Pseudocapacitors are another type of supercapacitors that use fast surface redox reactions for electrochemical storage. Therefore, pseudocapacitors can exhibit higher capacitance, but generally have a relatively short cycle life [148]. Recently, hybrid SCs have been proposed, offering higher energy density than EDLC without sacrificing cycling stability [149]. Electrode materials play a crucial role in determining electrochemical performance. And it is particularly important to explore electrode materials with high performance. The MXenes show high pseudocapacitive energy storage. However, the restacking and poor mechanical properties of MXenes limit its application.

**Figure 8:**
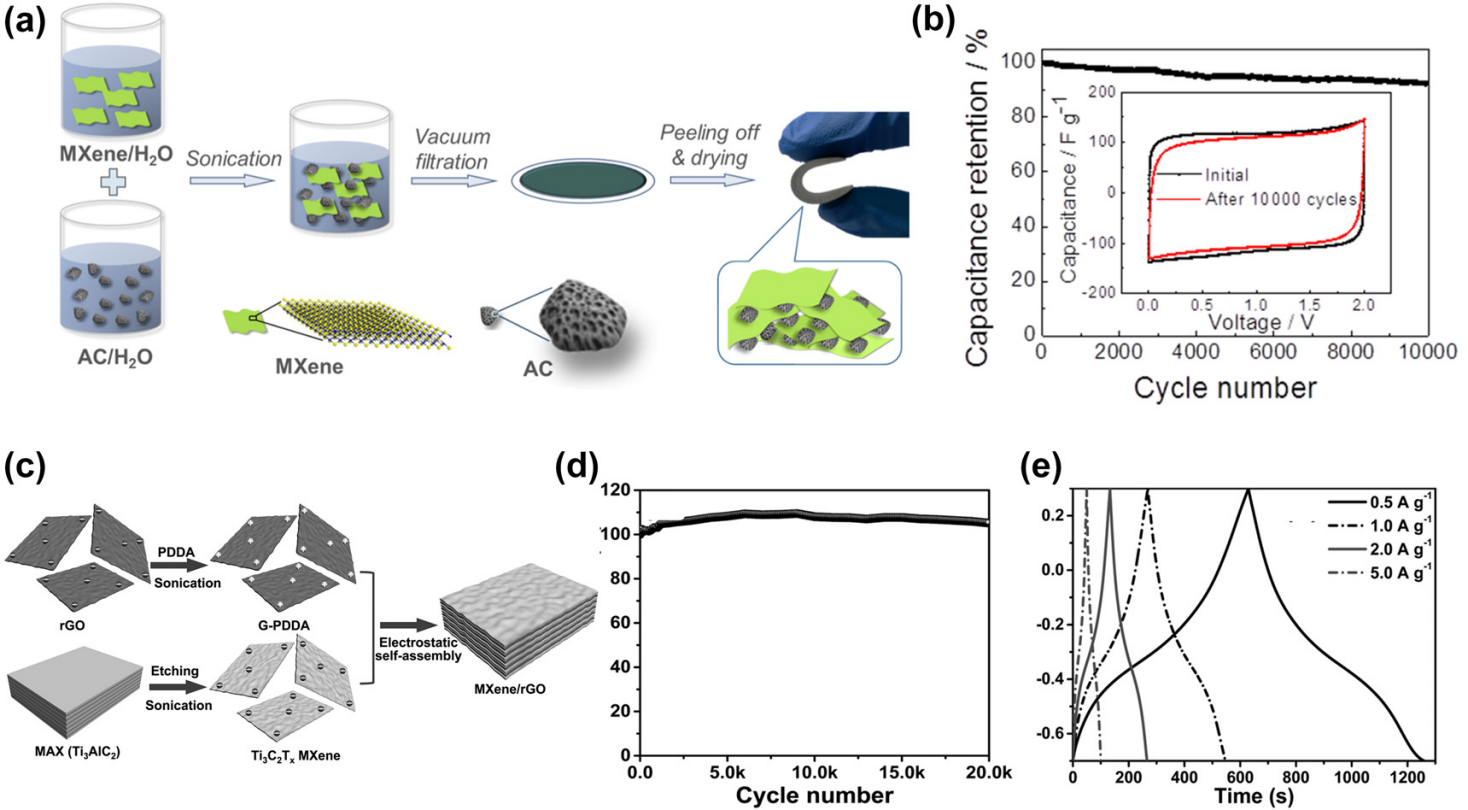
Preparation process, structure and electrochemical performance of MXene electrode for SCs. (a) Schematic diagram for the fabrication of MXene-bonded AC films, including mixing MXene flakes and AC particles in water, vacuum-assisted filtration, peeling off, and drying. (b) Cyclic performance at 10 A g^−1^. The inset is the CV curves at a scan rate of 20 mV s^−1^ at initial and after 10,000 cycles at 10 A g^−1^. Reproduced with permission [150]. Copyright 2018, American Chemical Society. (c) Schematic illustration for the synthesis of the MXene/rGO hybrids. (d) Cycling stability of the M/G-5% electrode measured at 100 mV s^−1^. (e) Galvanostatic charge/discharge curves of the M/G-5% electrode at different current densities. Reproduced with permission [48]. Copyright 2017, WILEY-VCH.

Carbon materials, such as activated carbon, graphene, and carbon nanotubes, show the advantages of high specific surface area and good stability. Combining Ti_3_C_2_T_*x*_ MXenes with these carbon materials can further improve the specific surface area and electrical conductivity of the composites. Yu et al. [150] fabricated a bendable Ti_3_C_2_T_*x*_ MXenes/AC composite by encapsulating activated carbon particles (AC) in MXenes layers as flexible electrodes for supercapacitors. MXenes play multifunctional roles in electrodes, including binders, flexible frameworks, conductive additives, and additional active materials. The Ti_3_C_2_T_*x*_ MXenes/AC composite electrode exhibited a high capacity of 126 F g^−1^ at 0.1 A g^−1^ and capacity retention of 92.4% after 100,00 cycles at 10 A g^−1^ (Figure 8(a) and (b)). Yu et al. designed MXenes/CNT hybrid fibers with an open helical structure by incorporating MXenes nanosheets into coiled CNT scaffolds in the corridor [151]. The hybrid structure can form more channel space to store electrolyte ions and ensure fast electron transport. The solid-state supercapacitor based on MXenes/CNT fiber exhibits an excellent capacity of 19.1 F cm^−3^ at 1.0 A cm^−3^, which is 31.8 times that of the bare CNT fiber-based device (0.59 F cm^−3^). Recently, Kim et al. fabricated MXenes/CNT electrodes by ion beam (FIB) technique [152]. The gap between the prepared MXene/CNT electrodes is 500 nm, enabling higher resolution. The MXenes/CNT electrode exhibits a high areal capacity of about 317 mF cm^−2^ at a scan rate of 50 mV s^−1^ and still maintains >30% capacity at 100 V s^−1^. Yan et al. [48] reported a simple method to fabricate MXenes/rGO flexible thin films via electrostatic self-assembly, as shown in Figure 8(c). The rGO nanosheets intercalated with MXenes layers to form well-ordered structures. The as-prepared MXenes/rGO electrode exhibits an excellent capacity of 1040 F cm^−3^ at a scan rate of 2 mV s^−1^. After 20,000 cycles, the as-synthesized composite exhibited good cycling performance with almost no capacity degradation (Figure 8(d)). From Figure 8(e), it can be found that the charge-discharge curves of the M/G-5% electrode are nonlinear, which is a typical feature of electric double-layer capacitors. A heterostructure composed of Ti_3_C_2_T_*x*_ MXenes and nitrogen-doped OMC (NOMC) layers was first reported by Allah et al. [153]. The Ti_3_C_2_T_*x*_-NOMC composite electrode exhibits a high gravimetric capacity of 329 F g^−1^ and a volumetric capacity of 823 F cm^−3^.

#### 3.2.2 Ti_3_C_2_T_*x*_/transition metal compound composites

**Figure 9:**
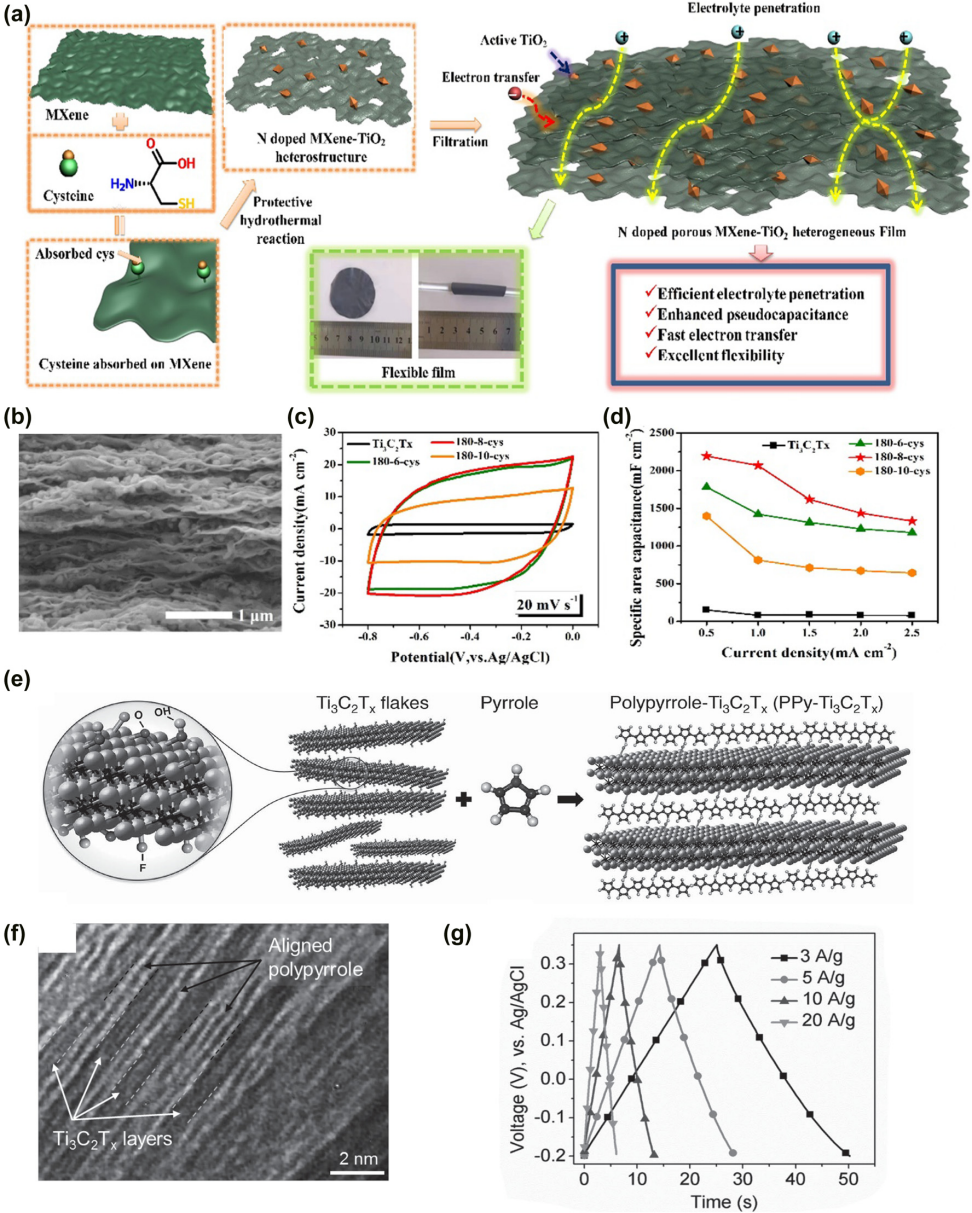
The structure and electrochemical performance of MXene composites electrode for SCs. (a) The schematic representation of the fabrication process of the nitrogen-doped porous MXene/TiO_2_ heterogeneous film. (b) SEM image of the cross-sectional view of the N-doped porous MXene/TiO_2_ film with high resolution. (c) Comparison of CV curves of different 180-x-cys samples in 1 M LiCl. (d) Comparison of specific areal capacitances of different 180-x-cys samples in 1 M LiCl; Reproduced with permission [154]. Copyright 2021, Elsevier B.V. (e) Schematic diagram of the synthesis of polypyrrole-Ti_3_C_2_T_*x*_. (f) TEM images of PPy/Ti_3_C_2_T_*x*_. (g) Galvanostatic charge/discharge curves of the PPy/Ti_3_C_2_T_*x*_ (1:2) film. Reproduced with permission [155]. Copyright 2015, Wiley-VCH.

Various metal/transition metal compound have been proposed as high-capacity electrode materials for supercapacitors, such as TiO_2_ [154], RuO_2_ [156], Co_3_O_4_ [157, 158], V_2_O_5_ [159], MnO_2_ [160–162], etc. [163]. The preparation of MXenes/metal oxide composites can be divided into *in-situ* preparation and *ex-situ* assembly. A nitrogen-doped porous MXenes/TiO_2_ heterostructure is rationally proposed by one-pot *in situ* polymerization [154]. The etched Ti_3_C_2_T_*x*_ MXenes colloidal suspension was stirred with an excess of cysteine (CYS) solution, absorbing CYS on thin MXenes layers. Then the hydrothermal treatment was used to obtain N-doped porous MXenes/TiO_2_ (Figure 9(a)). The electrical conductivity of N-doped MXenes can be well maintained, and the *in-situ* generated uniformly dispersed TiO_2_ can be uniformly inserted in MXenes layers, as shown in Figure 9(b). Figure 9(c) shows the cyclic voltammetry (CV) curves and GCD curves of the samples treated at 180 °C. The free-standing thin-film electrodes assembled based on N-doped porous MXenes/TiO_2_ heterolayers exhibit excellent energy storage performance with an outstanding specific capacity of 2194.33 mF cm^−2^ (918.69 F g^−1^), as shown in Figure 9(d). 3D Co_3_O_4_-doped MXenes/rGO hybrid porous aerogels were successfully prepared by *in-situ* reduction and thermal annealing processes [158]. The prepared composite showed a superior capacity of 345 F g^−1^ at 1 A g ^−1^ in 6 M KOH electrolyte, much higher than that of pure Ti_3_C_2_T_*x*_ MXenes, rGO, and MXenes/rGO electrodes. The application of two-dimensional transition metal dichalcogenides (TMDs) [164], metal-organic frameworks (MOFs) [163,165,166], etc., have shown excellent energy storage capacity through reversible surface redox reactions.

#### 3.2.3 Ti_3_C_2_T_*x*_/polymer composites

Although electrochemical polymers are widely used in flexible supercapacitor electrodes due to their high pseudocapacity and inherent flexibility, the structural instability and limited cycling stability of free-standing polymer films limit their applications. Ti_3_C_2_T_*x*_ MXenes can be combined with electrochemical polymers to enhance their energy storage capacity. Polypyrrole (PPy) has been successfully combined with Ti_3_C_2_T_*x*_ as a composite electrode for SCs. As shown in Figure 9(e), the resulting PPy/Ti_3_C_2_T_*x*_ composite exhibits a capacity of 1000 F cm^−3^ with capacity retention of 92% after 25,000 cycles. In Figure 9(g), the near-triangular galvanostatic charge-discharge curves confirm the high reversibility and good coulombic efficiency of the PPy/Ti_3_C_2_T_*x*_ composite electrode [155]. Zhu et al. [34] successfully intercalated PPy into I-Ti_3_C_2_T_*x*_ particles by electrochemical polymerization. The capacity of the PPy/I-Ti_3_C_2_T_*x*_ freestanding film reaches 203 mF cm^−2^. The cycling stability of the PPy/I-Ti_3_C_2_ film is greatly improved, with almost no capacitance loss observed after 20,000 charge/discharge cycles. Wu et al. [41] reported an approach to enhance the flexible cycling performance through the combination of PDT with layered Ti_3_C_2_T_*x*_ MXenes to form free-standing hybrid films. The thin-film electrode exhibits a high areal capacity of 284 mF cm^−2^ and low capacitance loss at 50 mA cm^−2^. Fu et al. [167] proposed a graphene-encapsulated Ti_3_C_2_T_*x*_ MXenes@polyaniline composite (GMP) which has a high gravimetric capacity of 635 F g^−1^ (volumetric capacity of 1143 F cm^−3^) at a current density of 1 A g^−1^ with excellent cycling stability of 97.54% after 10,000 cycles.

The layered structure of the 2D material provides a larger specific surface area, ensuring more exposure to the electrolyte. In summary, Ti_3_C_2_T_*x*_ MXenes materials and their composites with different structures show great potential as SC electrodes for high-performance energy storage. Although many significant achievements have been made, Ti_3_C_2_T_*x*_ MXenes -based materials are still a broad space for further exploration of SCs. In Table 2, we summarize the energy storage performance of different types of MXenes.

**Table 2:** The energy storage performances of different types of MXenes.

Materials	Type	Current density	Cycle number	Capacity	Ref.
Ti_3_C_2_T_*x*_ MXene	LIBs	0.5 C	650	723 mAh g^−1^	[80]
Fe_3_O_4_@Ti_3_C_2_	LIBs	5 C	800	280 mAh g^−1^	[81]
OMPDA/Ti_3_C_2_T_*x*_	LIBs	100 mA g^−1^	150	580 mAh g^−1^	[82]
3D porous MXene	LIBs	1 A g^−1^	3500	220 mAh g^−1^	[84]
s-MX@rGO	LIBs	10 A g^−1^	5000	241.5 mAh g^−1^	[85]
S/Ti_3_C_2_T_*x*_	LSBs	0.5 C	650	723 mAh g^−1^	[100]
N-Ti_3_C_2_T_*x*_/S	LSBs	2 C	1000	610 mAh g^−1^	[101]
S/PA-MXene/CNT	LSBs	0.5 C	800	712 mAh g^−1^	[96]
S@CTF/TNS	LSBs	1 C	1000	643.5 mAh g^−1^	[104]
3D Ti_3_C_2_T_*x*_ MXene	SIBs	2.5 C	1000	330 mAh g^−1^	[118]
Ti_3_C_2_/NiCoP	SIBs	1 A g^−1^	2000	261.7 mAh g^−1^	[122]
TiO_2_@Ti_3_C_2_T_*x*_	SIBs	960 mA g^−1^	5000	110 mAh g^−1^	[126]
MoSe_2_/MXenes@C	PIBs	200 mA g^−1^	100	355 mAh g^−1^	[134]
(GNF)/MXenes	PIBs	50 mA g^−1^	100	253.8 mAh g^−1^	[136]
MoO_3-x_/MXene	ZIBs	200 mA g^−1^	1600	369.8 mAh g^−1^	[145]
3D ZMO@Ti_3_C_2_T_*x*_	ZIBs	1 A g^−1^	5000	110 mAh g^−1^	[144]
Ti_3_C_2_T_*x*_ MXenes/AC	SCs	0.1 A g^−1^	10,000	126 F g^−1^	[150]
Co_3_O_4_-doped MXenes/rGO	SCs	1 A g^−1^	10,000	345 F g^−1^	[158]
Ti_3_C_2_T_*x*_/ZIF-67/CoV_2_O_6_	SCs	3 A g^−1^	4000	285.5 F g^−1^	[163]

### 3.3 Solar cells

#### 3.3.1 MXenes as an additive

**Figure 10:**
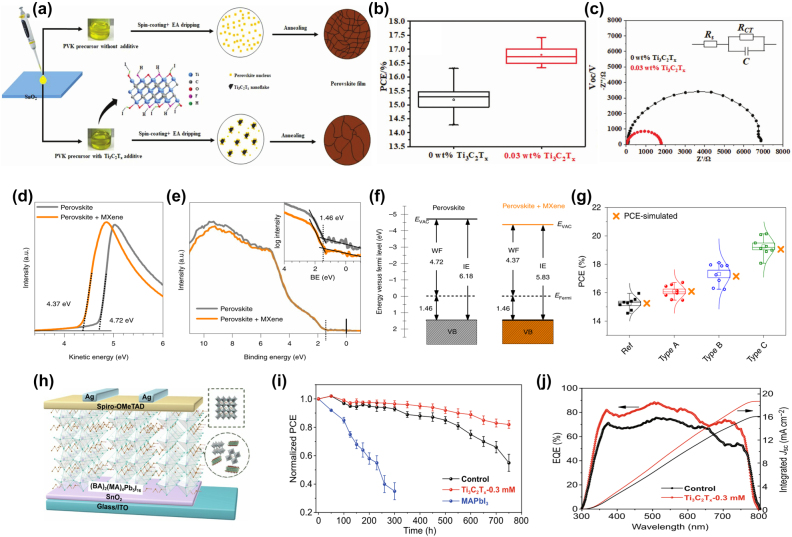
The preparation process and properties of MXene composites for perovskite solar cells. (a) Proposed nucleation and growth route of perovskite film with and without Ti_3_C_2_T_*x*_ additive. (b) The measured PCE of the device with and without 0.03 wt.% Ti_3_C_2_T_*x*_. (c) Nyquist plots of 0 and 0.03 wt% Ti_3_C_2_T_*x*_ additive–based device measured in the dark with a bias of 0.7 V. Reproduced with permission [169]. Copyright 2018, Wiley-VCH. UPS curves of pristine and MXene-doped perovskite films. (d)UPS spectra around the secondary electron cut-off. (e) UPS spectra in the valence band (VB) region. (f) Energy scheme for undoped and MXene-doped perovskite with respect to the EFermi. IE, ionization energy. EVAC, vacuum level. BE, binding energy. (g) Photovoltaic parameter statistics of PCE for the investigated PSCs. Reproduced with permission [170]. Copyright 2019, Springer Nature. (h) Schematic diagram of devices with the structure of Glass/ITO/SnO_2_/2D perovskite/Spiro-OMeTAD/Ag. (i) Stability of MAPbI_3_-based, control, and Ti_3_C_2_T_*x*_-doping devices without sealing at air atmosphere with humidity of 55 ± 5%. (j) EQE spectra and integrated Jsc of the control and optimized Ti_3_C_2_T_*x*_-doping devices. Reproduced with permission [171]. Copyright 2021, Springer Singapore.

Perovskite (PVSK) solar cells have developed rapidly in the past few years and have achieved many milestones in this field [40, 168]. In order to achieve higher PCE (30–33% in theory), some problems still need to be solved, especially the small grain size problem. Guo et al. [169] first proposed 2D Ti_3_C_2_T_*x*_ MXenes as an additive in PVSK solar cells. The results show that the groups of Ti_3_C_2_T_*x*_ MXenes can effectively delay the crystallization rate of CH_3_NH_3_PbI_3_, increasing the crystal size of CH_3_NH_3_PbI_3_ (Figure 10(a)). As shown in Figure 10(b), 0.03 wt.% Ti_3_C_2_T_*x*_ MXenes improve the average PCE parameter by 1.62%. In addition, the PVSK solar cells with Ti_3_C_2_T_*x*_ MXenes addition have smaller charge transfer resistance, as shown in Figure 10(c). This work opens up opportunities for the application of Ti_3_C_2_T_*x*_ MXenes as a potential material for perovskite solar cells.

Agresti et al. reported the introduction of MXenes into perovskite absorbers and TiO_2_ ETL to enhance the performance of PVSK solar cells [170]. The WF of pristine perovskite, determined from the secondary electron onset, is 4.72 eV which is shifted to 4.37 eV after the addition of MXenes (Figure 10(d)). The energy gap appears to be almost the same as the valence band spectrum of the original material (Figure 10(e)). As shown in Figure 10(f), the energy map shows the WF reduction of the MXenes-doped perovskite suppressed from 4.72 eV to 4.37 eV, while the ionization energy drops from 6.18 eV to 5.83 eV. Compared with the reference cell without Ti_3_C_2_T_*x*_ MXenes addition, the mixed MXenes-doped layer and MXenes-engineered interface exhibited a 26.5% improvement in cell efficiency, finally reaching 20.14% efficiency (Figure 10(g)). Jin et al. [171] used 2D Ti_3_C_2_T_*x*_ MXenes nanosheets with high electrical conductivity and high mobility as nano additives to fabricate perovskite films for PVSK solar cells (Figure 10(h)). Figure 10(i) demonstrated the device stability. The PCE of the solar cell increased from 13.69% to 15.71%, as shown in Figure 10(j).

#### 3.3.2 MXenes for hole/electron transport layer

**Figure 11:**
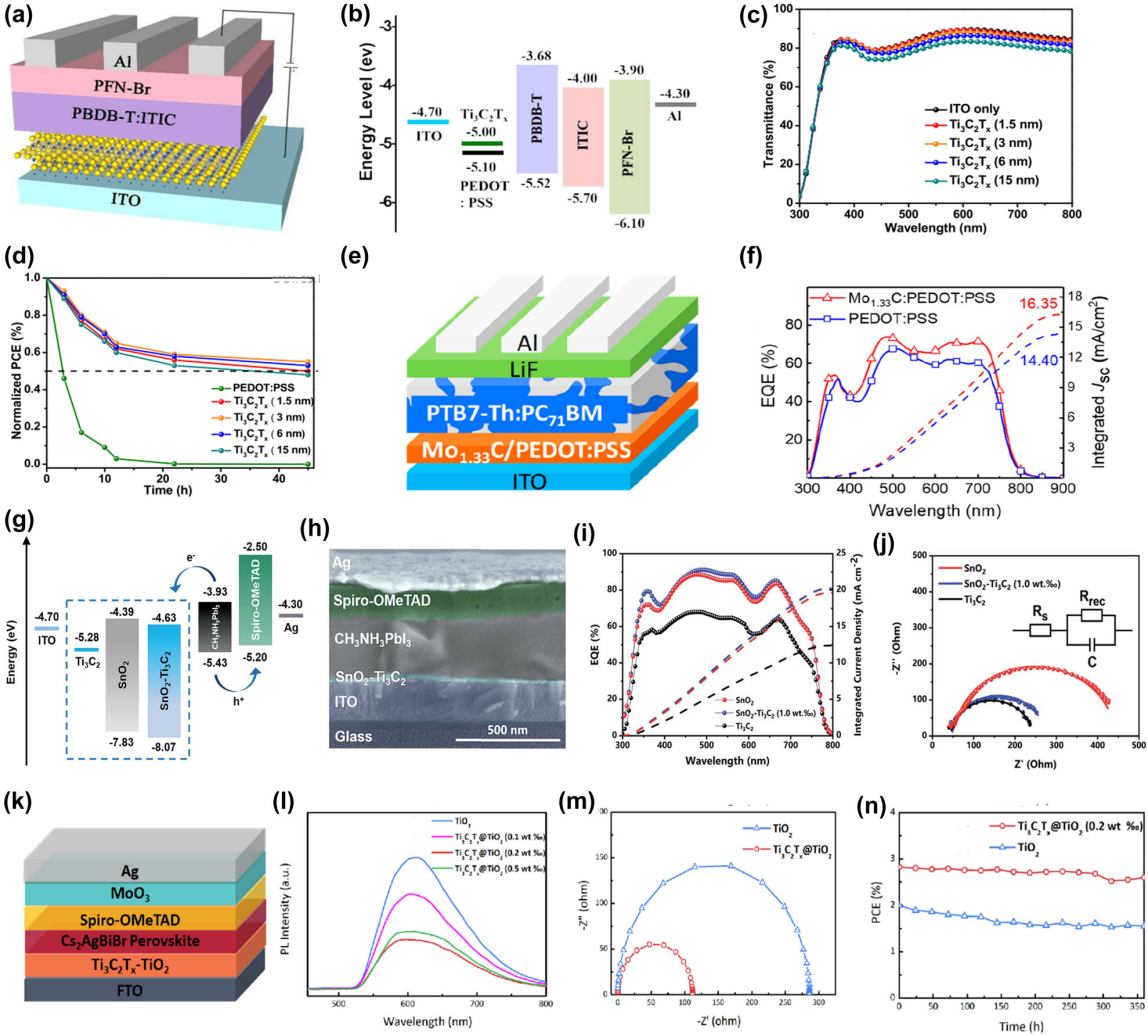
Schematic and properties of the PSC architecture. (a) Schematic of the PSC architecture. (b) Energy band diagrams of used materials. (c) Transmittance spectra of ITO, Ti_3_C_2_T_*x*_ (1.5 nm), Ti_3_C_2_T_*x*_ (3 nm), Ti_3_C_2_T_*x*_ (6 nm), and Ti_3_C_2_T_*x*_ (15 nm) films deposited on ITO substrates. (d) Stability of devices with different HTLs under atmosphere conditions without any encapsulation; Reproduced with permission [172]. Copyright 2019, Royal Society of Chemistry. (e) Schematic diagram of PSC structure and composition of the HTL. (f) EQE and integrated Jsc of PSCs using pristine and Mo_1.33_C-doped PEDOT: PSS as the HTL. Reproduced with permission [173]. Copyright 2020, Royal Society of Chemistry. (g) Schematic energy-level diagram of each layer. (h) Cross-sectional SEM image of the PSC device. (i) EQE spectra and the corresponding integrated current densities for the representative PSCs fabricated with SnO_2_, SnO_2_–Ti_3_C_2_ (1.0 wt%) and Ti_3_C_2_, respectively. (j) Nyquist plots of the PSCs with SnO_2_, SnO_2_–Ti_3_C_2_ (1.0 wt%), or Ti_3_C_2_ as ETLs under one sun illumination. Reproduced with permission [174]. Copyright 2019, Royal Society of Chemistry. (k) Schematic diagram of planar Cs_2_AgBiBr_6_ solar cell structure. (l) PL spectra of Cs_2_AgBiBr_6_ perovskite films deposited on Ti_3_C_2_T_*x*_@TiO_2_ and TiO_2_. (m) Nyquist plots of Ti_3_C_2_T_*x*_@TiO_2_ (0.2 wt %) and TiO_2_ based PSCs. (n) Ambient stability characteristics of the PSCs based on TiO_2_ and Ti_3_C_2_T_*x*_@TiO_2_ recorded under simulated AM 1.5 sunlight of 100 mW/cm^2^ irradiance. Reproduced with permission [175]. Copyright 2021, American Chemical Society.

For PSCs, the charge transport layer plays an important role in the photovoltaic performance and long-term device stability. Materials with high hole mobility, efficient electron and exciton blocking capabilities, good chemical stability and alignment of the valence band/HOMO level with the perovskite valence band are ideal HTL candidates for PSCs. Due to their unique optoelectronic properties, such as high carrier mobility, thickness-dependent band structure, ultra-thin thickness, and high compatibility with printable and flexible electronics, 2D material-based HTL can improve the photovoltaic performance and long-term stability of PSCs. Hou et al. used 2D Ti_3_C_2_T_*x*_ MXenes as hole transport layers (HTL) in polymer solar cells [172]. The Ti_3_C_2_T_*x*_ MXenes nanosheets were incorporated as HTL between the photoactive layer and the anode to facilitate the charge transport and collection properties in PSCs, as shown in Figure 11(a). According to Figure 11(b), hole transport is facilitated due to the matched energy arrangement of Ti_3_C_2_T_*x*_ and ITO. The PCE of this device is improved by 10.53%, which is significantly higher than that of pure ITO-based devices (4.21%). This photovoltaic performance also outperforms the state-of-the-art PEDOT: PSS-based device (10.11%). The optical properties of the HTL are crucial for the design of PSCs. To better evaluate the transport properties of Ti_3_C_2_T_*x*_ layers in PSCs, the transmittances of bare ITO and Ti_3_C_2_T_*x*_ thin films spin-coated with different thicknesses on ITO substrates were measured. The results show that the Ti_3_C_2_T_*x*_ film has good light transmittance Figure 11(c). In addition, the intercalated Ti_3_C_2_T_*x*_ HTL can effectively inhibit the corrosion of the active layer and ITO substrate by PEDOT:PSS compared with the PEDOT:PSS devices, and the Ti_3_C_2_T_*x*_-based devices also exhibit better long-term stability under atmospheric conditions, as shown in Figure 11(d). Liu et al. [173] reported MXenes mixed with PEDOT: PSS as HTL to realize high-performance polymer solar cells. The architecture of the device is shown in Figure 11(e). External quantum efficiency (EQE) and short-circuit current density (Jsc) were measured to evaluate device performance. As shown in Figure 11(f), both EQE and J_SC_ are enhanced compared to the device using PEDOT: PSS HTL. And the device with HTL composed of Mo_1.33_C mixed with PEDOT: PSS exhibits high PCE of 9.24%, while the PCE of the original device was 8.21%. Further studies revealed improvements in parameters, demonstrating that the hole transport properties of MXenes can improve overall device performance.

Besides HTL applications, MXenes are also used as ETLs to enhance the performance of solar cells [37]. MXenes can be candidates for transport layers in PSCs due to the good chemical stability, high electron mobility, and compatibility. Due to the unique electronic, optical, and plasmonic properties of MXenes, they have a wide range of potential applications. Yang et al. explored the application of Ti_3_C_2_T_*x*_ MXenes as ETL in organic-inorganic lead halide perovskite solar cells (PSCs) [174]. The schematic diagrams of the energy levels of each layer are shown in Figure 11(g) and (h). The SnO_2_–Ti_3_C_2_T_*x*_ MXenes layer acts as an effective agent, enabling matching energy level alignment between the ITO and CH_3_NH_3_PbI_3_ layers and increasing the conductivity of the ETL. By incorporating different Ti_3_C_2_T_*x*_ MXenes contents (0, 0.5, 1.0, 2.0 and 2.5 wt%), the best SnO_2_–Ti_3_C_2_T_*x*_ MXenes nanocomposites were screened. When doped with 1.0 wt% Ti_3_C_2_T_*x*_ MXenes, the SnO_2_–Ti_3_C_2_T_*x*_ MXenes nanocomposite can effectively increase the power conversion efficiency (PCE) from 17.23% to 18.34% while the PCE of the device fabricated using pristine Ti_3_C_2_T_*x*_ MXenes is only 5.28%. As shown in Figure 11(i), both EQE and integrated current density are enhanced. Electrochemical impedance spectroscopy (EIS) (Figure 11(j)) showed that Ti_3_C_2_T_*x*_ MXenes nanosheets provided a superior charge transfer path, enhanced electron extraction, electron mobility, and reduced electron transfer resistance at the ETL/perovskite interface, resulting in high photocurrent. Li et al. [175] adopted a simple strategy to dope monolayer MXenes nanosheets into titania (Ti_3_C_2_T_*x*_ MXenes@TiO_2_) and applied them as ETL in high-efficiency PVSK solar cells. The construction of this cell is shown in Figure 11(k). The single-layer MXenes nanosheets significantly improve the conductivity and electron extraction rate of TiO_2_. At the same time, the structure promotes the crystallization of Cs_2_AgBiBr_6_ double perovskite in solar cell devices. As shown in Figure 11(l), steady-state photoluminescence (PL) spectra were recorded. The photoluminescence quenching phenomenon shows that the fluorescence intensity of Cs_2_AgBiBr_6_ is significantly reduced after doping single-layer MXenes nanosheets which indicates that electrons are more efficiently extracted and transferred at the Ti_3_C_2_T_*x*_@TiO_2_/Cs_2_AgBiBr_6_ interface. The electrical conductivity of the device was further investigated by EIS, as shown in Figure 11(m), indicating that the high electrical conductivity of MXenes nanosheets can effectively reduce the electron transfer resistance and facilitate electron transfer. As shown in Figure 11(n), compared with pure TiO_2_-based devices, the PCE was improved by more than 40%, and the hysteresis is effectively suppressed.

#### 3.3.3 MXenes for flexible transparent electrodes (FTEs)

**Figure 12:**
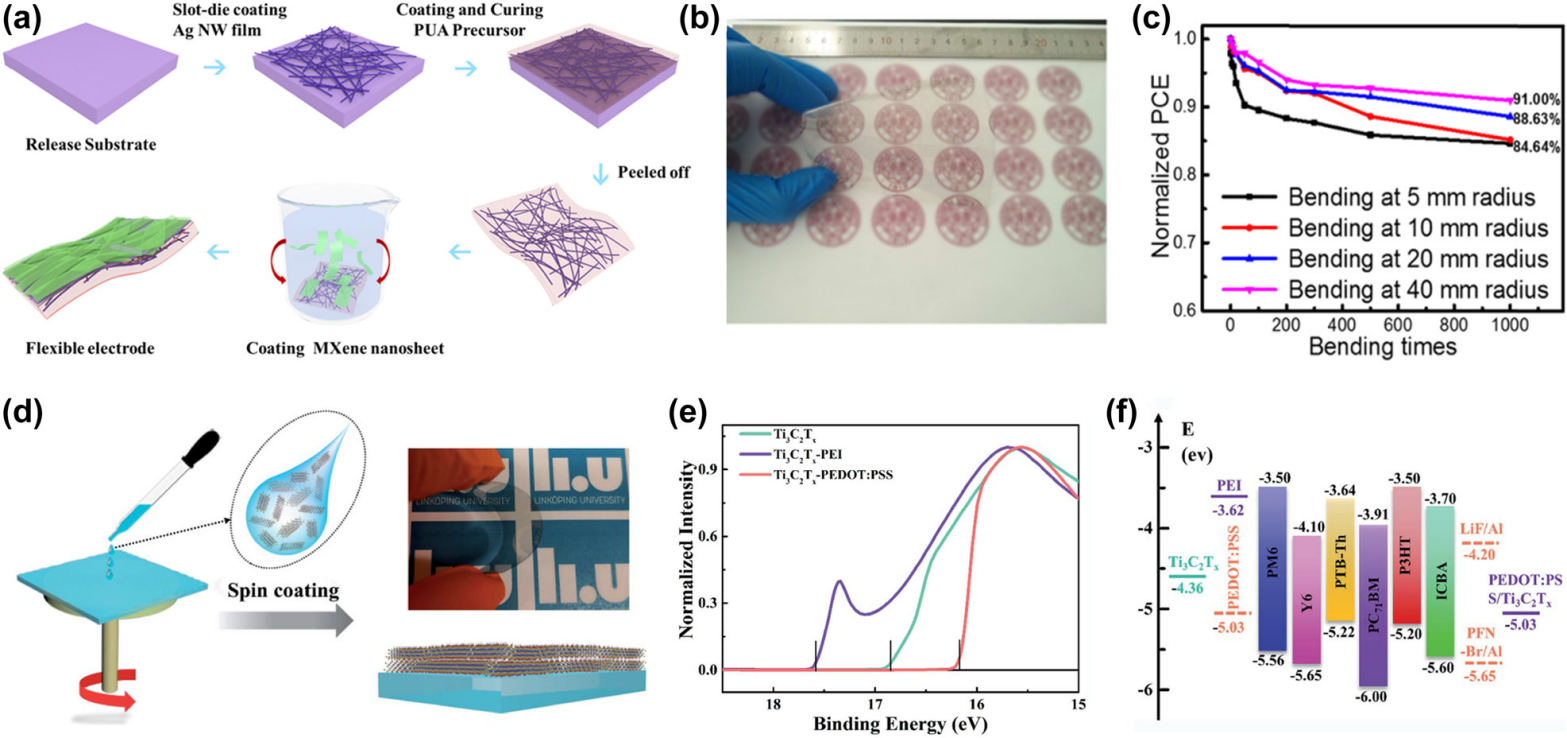
Fabrication process and properties of MXene-based flexible transparent electrode. (a) Fabrication Process of MXene-Based Flexible Transparent Electrode. (b) Optical images of the optimized MXene/AgNW-PUA films. (c) Normalized PCE of flexible PSCs with MXene/AgNW-PUA transparent electrodes at different bending radii as a function of the number of bending cycles. Reproduced with permission [176]. Copyright 2019, American Chemical Society. (d) Schematic of the preparation of a transparent flexible electrode. (e) Ultraviolet photoelectron spectroscopy (UPS) secondary electron cut-off region of Ti_3_C_2_T_*x*_ and Ti_3_C_2_T_*x*_ electrodes modified by PEDOT: PSS and PEI. (f) Energy level diagram of OPV devices. Reproduced with permission [177]. Copyright 2020, Royal Society of Chemistry.

In the past few years, much effort has been devoted to chemical doping and the design of flexible transparent electrodes (FTEs) for flexible organic solar cells (OSCs). MXenes and their hybrid electrodes emerge as the most promising flexible conductive materials, outperforming conventional indium tin oxides. Tang et al. combined Ag NW networks with MXenes nanosheets to fabricate flexible transparent electrodes for the first time and used this electrode to fabricate advanced flexible OPV (FOPV) devices [176]. The hybrid films were prepared by a simple and scalable solution processing method, as shown in Figure 12(a) and (b). The prepared hybrid films possessed high electrical conductivity, high transmittance and excellent mechanical properties. They demonstrated the ternary structure of PBDB-T: ITIC: PC71BM with a power conversion efficiency (PCE) of 8.30%. Bending tests showed that the flexible ternary organic solar cells were able to maintain 84.6% and 91% PCE of the original PCE after 1000 cycles at bending and unbending with bending radii of 5 and 40 mm, respectively (Figure 12(c)). Qin et al. [177] reported a Ti_3_C_2_T_*x*_ MXenes transparent electrode obtained by solution processing, as shown in Figure 12(d). Here, ultraviolet photoelectron spectroscopy (UPS) was used to probe the as-cast Ti_3_C_2_T_*x*_ MXenes films with poly(3,4-ethylenedioxythiophene): poly-(styrenesulfonate) (PEDOT: PSS) or polyethyleneimine. The WF values of the (PEI) modified Ti_3_C_2_T_*x*_ films are shown in Figure 12(e). Figure 12(f) shows the device architecture and the corresponding energy level diagram. In conclusion, MXenes exhibit extraordinary potential as flexible electrodes for solar cells and open new directions for next-generation transparent conducting electrodes.

### 3.4 Solar steam generations (SSG)

In the process of industrial production, the increasing shortage of fresh water resources has become a serious problem. Solar steam power generation utilizes inexhaustible solar energy to produce fresh water. It is considered to be an easy and effective way to solve the problem of water shortage. It uses photothermal materials to absorb the sun’s light through the photothermal conversion process and then converts it into heat energy for evaporation to produce fresh water. The evaporation efficiency (*η*) can be determined by the following equation

$$\eta =mh_{\text{LV}}/C_{\text{opt}}P_{0}$$

where *m* is the water evaporation rate, *h*_LV_ is the total enthalpy of liquid-vapor phase transition, *C*_opt_ is the optical concentration, and *P*_0_ is the nominal solar illumination of 1 Sun (1 kW m^−2^).

In order to achieve higher conversion efficiency, a reasonable evaporator should consider: (1) strong light absorption capacity and light-to-heat conversion efficiency; (2) low thermal conductivity to localize heat at the water-air interface; (3) high hydrophilic capacity and porous framework, allowing a constant supply of water to the absorber; (4) flotation capacity at the water/air interface. Currently, carbon materials [180], metal nanoparticles [181], transition metal dichalcogenides [182], hydrogels [178], etc., have been reported to achieve high solar steam efficiency. In recent years, the excellent electromagnetic wave absorption ability, tunable optical properties, and localized surface plasmon resonance of MXenes [181] have enabled them to exhibit excellent light-to-heat conversion capabilities, expanding the application in SSG. The applications of MXenes and their composites in the field of SSG are detailed.

**Figure 13:**
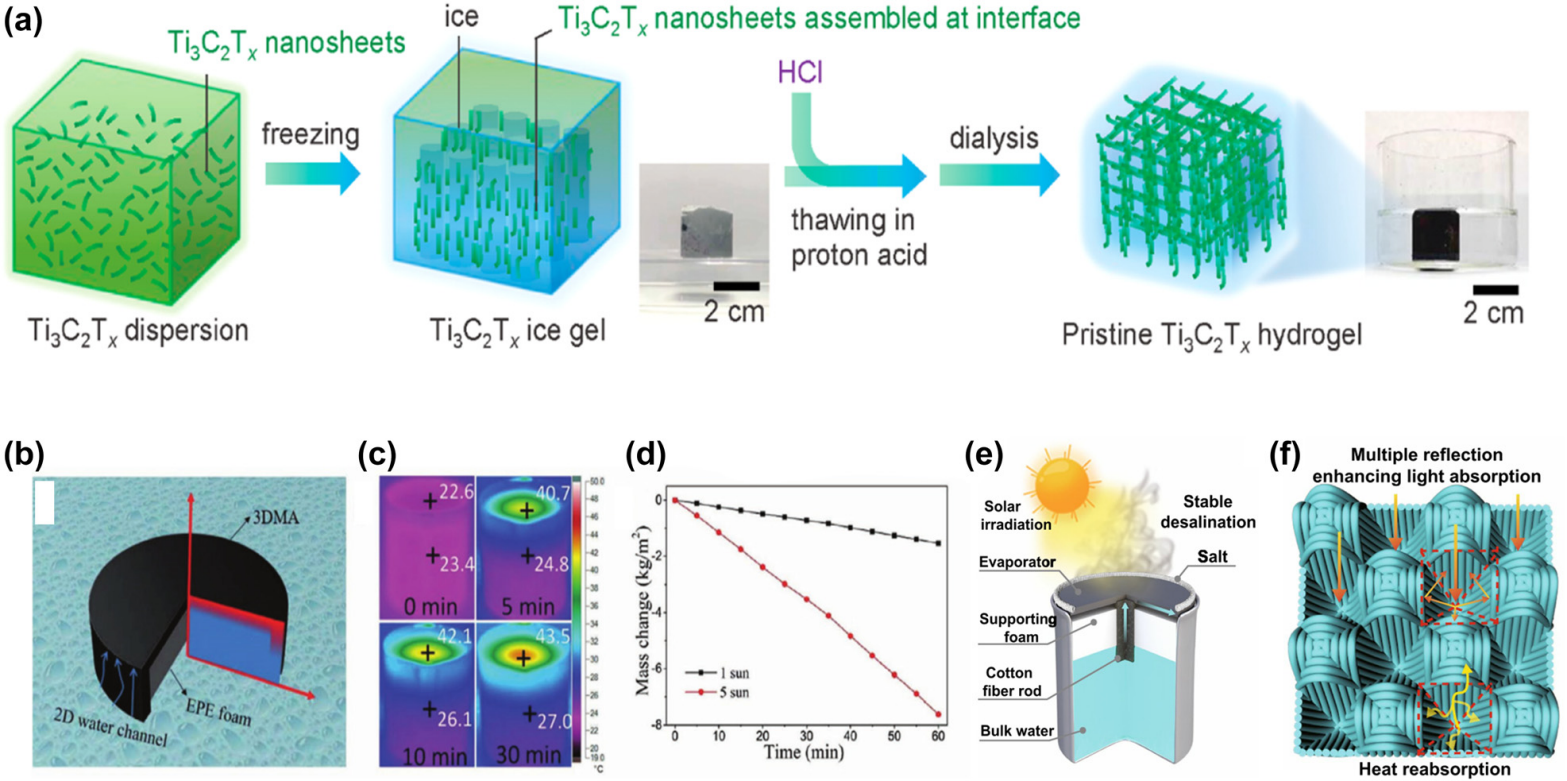
The structure and properties of Ti_3_C_2_T_x_ hydrogel. (a) Two-step approach to prepare the pristine Ti_3_C_2_T_*x*_ hydrogel, which includes ice-template preassembly and thawing in HCl solution. Reproduced with permission [178]. Copyright 2020, American Chemical Society. (b) Schematic illustration of the 3DMAs embedded with EPA foam as the thermal insulation layer. (c) Infrared images of water and 3D MA surfaces under 1 Sun illumination of 0, 5, 10, and 30 min. (d) The mass change of water for 3DMA with EPA foam under the solar illumination of 1 Sun and 5 Sun, respectively. Reproduced with permission [179]. Copyright 2019, Royal Society of Chemistry. (e) Schematic diagram of MXene-decorated 3D honeycomb fabric-based solar evaporator for desalination. (f) Schematic illustration of the 3D honeycomb-structured fabric for light absorption and heat reabsorption. Reproduced with permission [75]. Copyright 2021, The Author(s).

Li et al. [183] reported the application of 2D MXenes for solar steam power generation. Ti_3_C_2_T_*x*_ MXenes possess nearly 100% internal light-to-heat conversion efficiency. Furthermore, a clear high-temperature region (35.4 °C) can be observed at the air/water interface, which indicates that water heating is enabled at the interface and provides strong evidence. Polystyrene (PS) foam is used as thermal insulation material, and the solar-water conversion efficiency reaches 84% under 1 sun illumination. In Ti_3_C_2_T_*x*_ MXenes, replacing Li^+^ with protons can enhance the interaction between Ti_3_C_2_T_*x*_ MXenes nanosheets. The deintercalation of Li^+^ is crucial to preventing complete solvation of Ti_3_C_2_T_*x*_ MXenes nanosheets and decomposition of Ti_3_C_2_T_*x*_ MXenes assemblies. Based on this intercalation chemistry, Chen et al. [178] fabricated pristine Ti_3_C_2_T_*x*_ MXenes hydrogel matrices with arbitrary microstructures using freeze-induced pre-assembly and a thawing process in protonic acid (Figure 13(a)). In the absence of other external components, the material properties of the as-synthesized pristine Ti_3_C_2_T_*x*_ MXenes hydrogels reached their maximum. The compressive modulus is 2.4 MPa and the electrical conductivity is 220.3 ± 16.8 S/m when the solid content was 5%. Anisotropic Ti_3_C_2_T_*x*_ MXenes hydrogels facilitate fast water transport within vertical microchannels, bringing a competitive performance in solar steam generation (1.90 kg m^−2^ h^−1^ under 1 kW m^−2^ irradiation). Zhao et al. [179] reported solar steam generators of 3D MXenes frameworks (3DMAs) by assembling MXenes nanosheets onto melamine foam (MF) frameworks. Compared with 2D MXenes, the high porosity of 3DMAs enables them to float on water (Figure 13(b)). The macroporous structure of MF can provide channels for water transport, and the hydrophilic properties of MXenes can fully wet the internal framework. Therefore, the water supply can be continued. 3DMAs exhibit efficient broadband solar absorption (∼98%) and excellent solar heat conversion capability. Under 1 sun illumination, the solar steam efficiency is as high as 88.7%, and the water evaporation rate is as high as 1.409 kg m^−2^ h^−1^ (Figure 13(d)). In addition, polyethylene (EPE) foam is embedded as insulation to reduce heat loss to the bottom water. Therefore, after 30 min of irradiation, the temperature of the 3DMAs system reached to 43.5 °C while the bulk water was still around room temperature (Figure 13(c)). The results show that the 3D morphology of the composites can provide guidance for the effective design of interfacial solar steam power generation systems. Lei et al. [75] obtained inspiration from nature and innovatively designed a 3D honeycomb-like fabric decorated with hydrophilic Ti_3_C_2_T_*x*_ MXenes as a solar evaporator (Figure 13(e) and (f)). 3D MXenes honeycomb fabrics were prepared by impregnating positively charged poly (diallyldimethylammonium chloride) (PDDA) in a negatively charged MXenes dispersion. The 3D honeycomb fabric has a rough surface with high porosity and a well-aligned honeycomb concave structure that creates light traps through scattering and omnidirectional light absorption for maximum light capture. In addition, the construction of thermal insulation barriers connected to the 1D waterway and the rational recovery of convective and radiant energy enable localized photothermal generation to achieve the goal of achieving minimal heat loss. The solar efficiency of the 3D honeycomb fabric evaporator reaches 93.5% under one solar irradiation, and the evaporation rate is 1.62 kg m^−2^ h^−1^.

**Figure 14:**
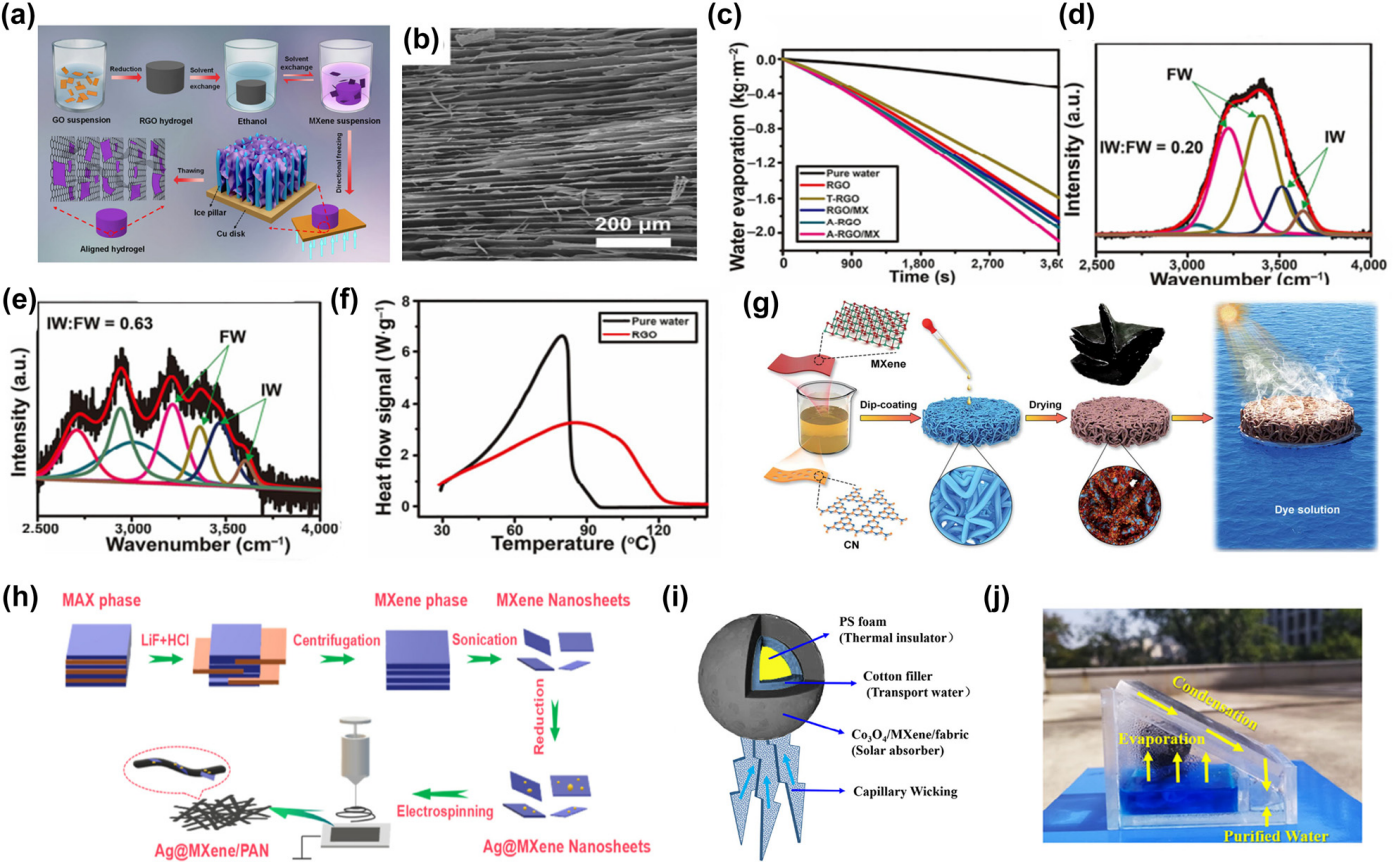
The fabrication and properties of A-RGO/MX hybrid hydrogel. (a) Schematic illustration of the fabrication of A-RGO/MX hybrid hydrogel. (b) SEM images of freeze-dried A-RGO/MX hydrogel. (c) Mass changes of water without or with RGO, T-RGO, A-RGO, and A-RGO/MX hydrogels under 1-sun irradiation. (d) Without and (e) with the RGO hydrogel, where FW and IW represent free water and intermediate water, respectively. (f) Thermograms of pure water and RGO hydrogel. The magnitudes of DSC signals are proportional to the heat flow during the measurement. Reproduced with permission [184]. Copyright 2020, Springer Nature. (g) Scheme for preparing the CNMC-x hybrid membrane via dip-coating the mixture of CN and the MXene in PVA solution on the surface of non-woven cotton cloth for efficient solar distillation of contaminated water and photocatalytic degradation of organic pollutants in the contaminated water. The inset shows the photograph of the folded CNMC-5 hybrid membrane to show its flexibility. Reproduced with permission [185]. Copyright 2021, The Royal Society of Chemistry. (h) Schematic diagram of the fabrication of Ag@MXene/PAN nanofiber membrane. Reproduced with permission [186]. Copyright 2021, Elsevier Ltd. (i) The biomimetic architectural structure of 3D spherical evaporator. (j) Photograph of a solar-driven evaporation device for outdoor practical applications. Reproduced with permission [187]. Copyright 2020, WILEY-VCH.

In addition, nanocarbon materials such as carbon nanotubes [180], graphene [144, 184], etc., can be well combined with MXenes to improve the generation rate of solar steam. Li et al. [184] used a directional freezing technique to fabricate vertically aligned reduced graphene oxide (A-RGO) hydrogels for efficient solar steam production (Figure 14(a) and (b)). Hydrophilic Ti_3_C_2_T_*x*_ MXenes nanosheets were coated in the A-RGO hydrogel framework to generate vertically aligned RGO/MXenes (A-RGO/MX) hybrid hydrogels. The hybrid hydrogel exhibits an average water evaporation rate of 2.09 kg m^−2^ h^−1^ under a single solar illumination (Figure 14(c)) with a high conversion efficiency of 93.5%.

They also analyzed the effect of the A-RGO/MX composite hydrogel on the evaporation enthalpy and water evaporation rate. The molar ratio of intermediate water to free water in the A-RGO hydrogel was calculated to be 0.63, which is approximately 3.1 times that of pure water (0.20) (Figure 14(d) and (e)). Evaporation energy of pure water and water in hydrogels was measured using differential scanning calorimetry (DSC) (Figure 14(f)) to demonstrate the reduction in the enthalpy of evaporation of water in hydrogels. Ding et al. [185] prepared a synergistic photothermal-photochemical hybrid film by dip-coating 3D porous carbon nitride (CN) and 2D MXene on nonwoven fabrics (Figure 14(g)). Under simulated sunlight, there is high light absorption across the entire solar spectrum. And it has good photothermal conversion, less non-radiative reflection loss and increased photocurrent. The water evaporation rate can reach 2.30 kg m^−2^ h^−1^, and the solar-steam conversion efficiency is 98.9% under one solar irradiation. They built a large-scale solar energy conversion unit outdoors with a daily freshwater production of 5.7 kg m^−2^, which can meet the needs of two adults.

Metal nanoparticles (NPs) usually have a unique localized surface plasmon resonance (LSPR) effect [181]. It refers to the fact that when light is incident onto metal nanoparticles if the incident photon frequency matches the overall vibration frequency of the metal nanoparticles conducting electrons, the nanoparticles show a strong absorption effect on the photons. The LSRP effect induces the generation of photothermal electrons, which is beneficial to the absorption of light, especially in the near-infrared region. The absorbed solar energy is converted into heat, which is dissipated into the surrounding medium by vibrations scattered by the lattice, thereby increasing the surrounding temperature. As shown in Figure 14(h), Liu et al. [186] reported silver nanoparticle-decorated MXenes nanosheets/polyacrylonitrile (Ag@MXenes/PAN) nanofibers as vaporizers by functionalizing MXenes nanosheets with silver nanoparticles using electrospinning technology and origami process. The combination of Ag nanoparticles with MXenes nanosheets not only enhances broadband light absorption and thermal yield, but also contributes to its good sunlight capture, catalysis, and antibacterial capabilities. In addition, the Ag@MXenes/PAN nanofibrous membrane exhibits excellent flexibility and foldability. The 3D origami-type structure evaporator with high evaporation surface area and stronger light absorption can be rationally designed. The evaporation capacity of this evaporator under 1 sun irradiation reaches 2.08 kg m^−2^ h^−1^, which is one of the most advanced solar evaporators based on electrospinning nanofibers. In addition, various metal oxides or sulfides, such as SiO_2_, Co_3_O_4_, MoS_2_, etc., hybridized with MXenes have been proposed as solar evaporators to enhance the steam evaporation rate. Li et al. [188] coated MXene nanosheets and low thermal conductivity SiO_2_ on hydrophilic poly (tetrafluoroethylene) (HPTFE) membranes by commercial continuous spraying to obtain the SiO_2_/MXene/HPTFE Janus membrane. They have excellent mechanical properties, high stability and light absorption of 93.0%. The photothermal evaporation rate is 1.53 kg m^−2^ h^−1^, the solar photothermal conversion efficiency is 85.6%, and the removal rate of wastewater and organic pollutants can reach 99.9%. Lu et al. [187] anchored Co_3_O_4_ nanoparticles on layered ultrathin MXene nanosheets to construct an integrated Co_3_O_4_/Ti_3_C_2_ MXene-based fabric 3D spherical evaporator. As shown in Figure 14(i) and (j). The evaporation rate reaches 1.89 kg m^−2^ h^−1^, and the light energy-steam energy conversion efficiency exceeds the theoretical limit by up to 130.4%. In Table 3, we summarize the energy conversion performances of different MXenes.

**Table 3:** The energy conversion performances of different MXenes.

Photothermal materials	Evaporation rate (kg m^−2^h^−1^)	Efficiency (%)	Solar intensity (sun)	Ref.
Ti_3_C_2_T_*x*_ MXene	1.90	97	1	[178]
3D MXenes	1.409	88.7	1	[179]
3D MXenes honeycomb fabric	1.62	93.5	1	[75]
A-RGO/MX	2.09	93.5	1	[183]
SiO_2_/MXene/HPTFE	1.53	85.6	1	[187]
Co_3_O_4_/Ti_3_C_2_ MXene	1.89	130.4	1	[186]
Ag@MXenes/PAN	2.08	93	1	[185]
MXene@MoS_2_	1.39	91	1	[182]

Although the 100% internal light-to-heat conversion efficiency of Ti_3_C_2_T_*x*_ MXenes, the weak wettability limits light due to hydrophobic modification and insufficient water transport channels. Regarding the potential applications of MXenes in seawater desalination and wastewater purification, it is still necessary to further explore their solar steam efficiency improvement strategies.

## 4 Conclusions

In this review, we firstly introduce preparation methods for MXenes-based flexible materials. Secondly, the application progress of MXenes and MXenes-based nanocomposites in the fields of MXenes-based energy storage and conversion applications including lithium-ion batteries, lithium-sulfur batteries, sodium-ion batteries, supercapacitors, solar cells, and solar steam generation is introduced. Finally, the effects of MXenes properties and interlayer structure on the energy storage and energy conversion efficiencies are discussed. The unique 2D layered structure and excellent electrical conductivity of MXenes, as well as the fast ion/electron transfer rate and enhanced structural stability, guarantee its use as a conductive substrate in various energy storage devices.

MXenes are active materials exhibiting pseudocapacitive behavior in metal-ion batteries and supercapacitors because their abundant surface functional groups provide sufficient active sites for fast surface redox reactions. In addition, due to the high ion diffusion mobility and low ion adsorption energy of MXenes, it can also be used as an electrode material for various metal batteries to achieve a higher stability and battery capacity. Likewise, the electrochemical performance can be significantly improved when MXenes-based is mixed with other advanced materials such as carbon materials, polymers, and TMCs to create synergistic heterojunctions [189]. MXenes possess high charge carrier mobility, high electrical conductivity, and high optical transparency, as well as excellent electromagnetic wave absorption, tunable optical properties, and localized surface plasmon resonance effects. The work function can be effectively modified in solar cells, the interfacial properties, electron mobility and synergistic effect are significantly enhanced, and the power conversion efficiency and stability can be greatly improved. It exhibits excellent photothermal conversion ability in the field of SSG and can achieve higher conversion efficiency.

Although MXenes are considered to be promising candidates for energy storage and conversion applications, there are still several challenges: Firstly, the synthesis method of MXenes with a precise number of layers, enhanced interlayer spacing, programmable surface groups and controllable atomic defects are not well obtained. The exfoliation of multilayered Ti_3_C_2_T_*x*_ and subsequent collection may be further developed. The existing surface terminations (such as –O, –OH, –F) can affect the structural, electronic, magnetic, and other properties of Ti_3_C_2_T_*x*_, and thus, this interrelation between functional groups and Ti_3_C_2_T_*x*_ properties is remained to be understood. Secondly, the ionic dynamics and charge storage mechanisms between MXenes nanosheets are still unclear, which is important to achieve excellent performance. The exploitations of Ti_3_C_2_T_*x*_-based hybrid structures, such as layer-by-layer, cross-linked, insertion, and anchoring, still require more efforts. Thirdly, detailed interfacial studies and stability studies are required to obtain outstanding optoelectronic properties. Further research is needed to gain a deeper understanding of the effects of photothermal conversion. Finally, the mass production of MXenes remains a challenge for the practical fabrication of high-performance solar cells. The increasing investigations are expected to address these challenges to further promote the application of Ti_3_C_2_T_*x*_ MXenes-based flexible materials in electrochemical energy storage and solar energy conversion.
